# Proteins that physically interact with the phosphatase Cdc14 in *Candida albicans* have diverse roles in the cell cycle

**DOI:** 10.1038/s41598-019-42530-1

**Published:** 2019-04-18

**Authors:** Iliyana N. Kaneva, Ian M. Sudbery, Mark J. Dickman, Peter E. Sudbery

**Affiliations:** 1 ChELSI Institute, Department of Chemical and Biological Engineering, University of Sheffield, Sheffield, S1 3JD UK; 2 Department of Molecular Biology and Biotechnology, University of Sheffield, S10 2TN Sheffield, UK; 3 Present Address: MRC London Institute of Medical Sciences, Biological Mass Spectrometry & Proteomics, CRB Building, Hammersmith Hospital Campus, Du Cane Road, London, W12 0NN UK

**Keywords:** Cell-cycle exit, Molecular biology

## Abstract

The chromosome complement of the human fungal pathogen *Candida albicans* is unusually unstable, suggesting that the process of nuclear division is error prone. The Cdc14 phosphatase plays a key role in organising the intricate choreography of mitosis and cell division. In order to understand the role of Cdc14 in *C*. *albicans* we used quantitative proteomics to identify proteins that physically interact with Cdc14. To distinguish genuine Cdc14-interactors from proteins that bound non-specifically to the affinity matrix, we used a substrate trapping mutant combined with mass spectrometry analysis using Stable Isotope Labelling with Amino Acids in Cell Culture (SILAC). The results identified 126 proteins that interact with Cdc14 of which 80% have not previously been identified as Cdc14 interactors in *C*. *albicans* or *S*. *cerevisiae*. In this set, 55 proteins are known from previous research in *S*. *cerevisiae* and *S*. *pombe* to play roles in the cell cycle, regulating the attachment of the mitotic spindle to kinetochores, mitotic exit, cytokinesis, licensing of DNA replication by re-activating pre-replication complexes, and DNA repair. Five Cdc14-interacting proteins with previously unknown functions localised to the Spindle Pole Bodies (SPBs). Thus, we have greatly increased the number of proteins that physically interact with Cdc14 in *C*. *albicans*.

## Introduction

*Candida albicans* is normally a harmless commensal of the skin, urogenital and gastrointestinal tracts. However, in otherwise healthy individuals it can be responsible for debilitating and recurrent mucosal infections. In immunocompromised and other classes of vulnerable patients it causes life-threatening bloodstream infections^1–3^. It is normally diploid and lacks the capability to undergo meiosis. Although lacking a sexual cycle, *C*. *albicans* can undergo a parasexual cycle in which two diploid cells mate to form a tetraploid, which then sheds chromosome during subsequent mitotic divisions to regain either diploid or aneuploidy states^4^. Stresses, such as exposure to the antifungal drug fluconazole, result in ploidy changes, loss of heterozygosity and whole chromosome and segmental aneuploidy (reviewed in^5–8^). Such genome plasticity is thought to be a major generator of diversity in the absence of a sexual cycle and has been shown to be adaptive. Recently, in response to fluconazole or passage through mice it has been shown that diploids can be reduced by chromosome loss to generate mating-competent haploids^9^. This genome plasticity occurs through non-disjunction events in mitosis, which implies a high error rate in the intricately choreographed events of mitosis.

A key player in orchestrating mitosis and cell division is Cdc14, a dual specificity, proline- and serine-directed phosphatase (for reviews see^10–12^). In the budding yeast *Saccharomyces cerevisiae* after activation by the Fourteen Early Anaphase Release (FEAR) and Mitotic Exit Network (MEN) pathways it ensures irreversible exit from mitosis by directing the destruction of G2 cyclins and disassembly of the spindle. After mitotic exit it relocates to the bud neck and activates cytokinesis. Finally, in the G1 of the next cycle it reactivates DNA origins, which were inactivated after firing in the previous cell cycle^13^. Previously, *Ca*Cdc14 has been shown not to be essential for viability, but it is required for normal polarized growth of hyphae and regulation of *Ca*Cdc14 has been shown to be necessary for the inhibition of cell separation characteristic of hyphal growth^14^.

Given the central role of Cdc14 in orchestrating mitosis and the key role of non-disjunction in this pathogen, it is important to investigate the way Cdc14 operates in *C*. *albicans*. A key objective in studying the role of a kinase or a phosphatase is to assemble list of its targets, which in turn requires the identification of proteins with which it physically interacts. In this study, we have used a substrate trapping mutant of Cdc14^15,16^ in conjunction with affinity purification (AP) mass spectrometry (MS) analysis. We have used Stable Isotope Labelling with Amino Acids in Cell Culture (SILAC) in conjunction with quantitative MS analysis to readily distinguish physically-interacting partners against a large number of background proteins^17–20^. This powerful approach has led to new insights into the involvement of Cdc14 in the cell cycle in *C*. *albicans*.

## Results

### Generation and characterization of a substrate trapping mutant of Cdc14

**Figure 1:**
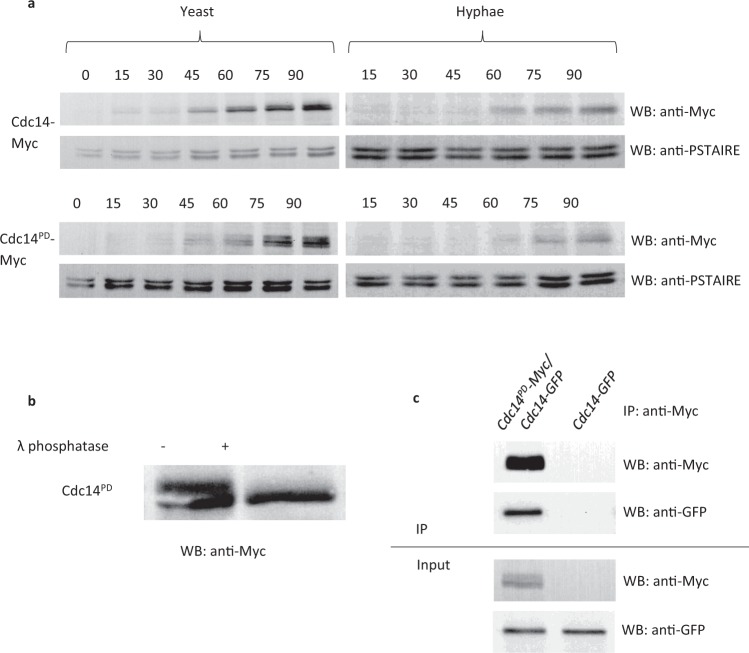
Characterisation of Cdc14^PD^. (**a**) Time course of Cdc14^PD^-Myc expression. *C*. *albicans* cells were grown overnight and then starved in water for 4 hours to induce transition into G0. They were then released into fresh medium and left to grow in either yeast- or hyphae-promoting conditions. Aliquots were removed every 15 min and processed for Western blotting using an anti-Myc monoclonal antibody. An anti-PSTIARE antibody that recognises Cdk1 and Pho85 was used as a loading control. The phosphatase is not present in stationary phase cells and it starts appearing after about 45 min in yeast and 60 min in hyphae. The temporal pattern of Cdc14-Myc and Cdc14^pd^-Myc expression were similar. Both Cdc14-Myc and Cdc14^PD^-Myc are expressed at lower levels in hyphae. Note the presence of a retarded band in Cdc14^PD^-Myc showing increased phosphorylation compared to Cdc14-Myc. (**b**) Treatment of the lysate from *C*. *albicans* cells expressing Cdc14^PD^-Myc with λ phosphatase results in the disappearance of the retarded band showing the protein is phosphorylated. Full length autoradiograms are presented in Supplementary Fig. S1. (**c**) Cdc14^PD^-Myc immunoprecipitates Cdc14-GFP showing a physical interaction. Cell lysates were immunoprecipitated with αMyc (IP) and the resulting Western blot probed with αMyc or αGFP as indicated.

Previous studies have shown that the mutation in *S*. *cerevisiae* Cdc14C283S is inactive and substrate trapping^16^. C275 in *Ca*Cdc14 was identified as the corresponding residue by alignment, so we generated *Ca*Cdc14C275S as a phosphatase dead (PD), substrate trapping mutant of *Ca*Cdc14, which will be referred to here as Cdc14^PD^. We generated *CDC14/cdc14*^PD^-*MYC*, and *CDC14/cdc14*^PD^-GFP strains. Both strains had wild type morphology in yeast and hyphal growth forms showing the *cdc14*^*-PD*^ allele is recessive to the wild type allele. Expression levels of Cdc14-Myc and Cdc14^PD^-Myc were compared through the cell cycle of *C*. *albicans* yeast and hyphae by Western blot (Fig. 1a). Expression of both Cdc14 and Cdc14^PD^ was low in G1 and increased after 45 minutes in yeast cells and 60 minutes in hyphal cells, consistent with previous observations^14^. However, we also observed that expression of both the wild type and mutant proteins were lower in *C*. *albicans* hyphae compared to yeast. Such differential expression was also evident in a previous report^14^. Cdc14^PD^-Myc was phosphorylated, as indicated by the band shift seen on a Western blot, which disappeared upon phosphatase treatment (Fig. 1b). In contrast, Cdc14-Myc did not show phosphorylation. Clp1 (the *S*. *pombe* orthologue of CaCdc14) is subject to autodephosphorylation in *S*. *pombe*^21^ and we have found CaCdc14^PD^*-*Myc physically interacts with Cdc14-GFP (Fig. 1c) suggesting this may also be the case in *C*. *albicans*. In order to verify that the Cdc14^PD^ allele is non-functional we placed the remaining functional copy of *CDC14* under the control of the regulatable *MET*3 promoter^22^ (*MET3-CDC14/cdc14*^*PD*^*-MYC*). Under *MET*3-repressing conditions, hyphal cells showed morphological defects characteristic of the *cdc14*Δ/Δ mutants in *C*. *albicans*^14^, confirming that Cdc14^PD^ is non-functional (Supplementary Fig. S2).

### Identification of Cdc14^PD^ interacting proteins

**Figure 2:**
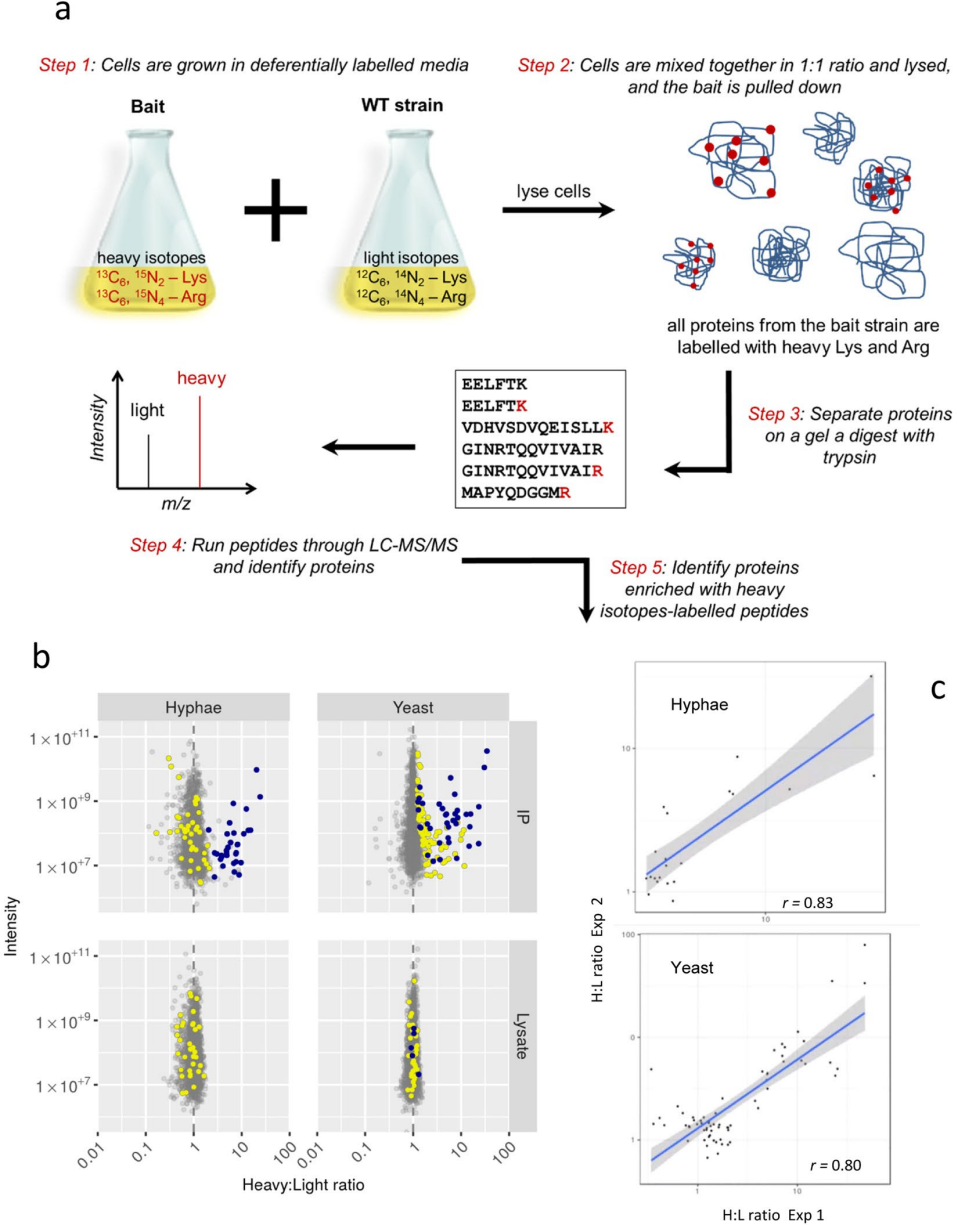
Identification of Cdc14 interactors using SILAC. (**a**) Experimental strategy. *CDC14-/ MET3-cdc14*^PD^–*Myc* cells were grown in depressing heavy medium alongside parental untagged cultures grown in light medium and the lysates mixed in equal quantities prior to mass spectrometry analysis. Cdc14^PD^–Myc was immunoprecipitated and the immuneprecipitate analysed using a quadrupole Orbitrap-MS. Peptides from genuine interacting proteins will show a significant deviation from 1:1 H:L ratio. (**b**) Volcano plot of intensity versus H:L ratio of detected proteins. Blue dots show proteins enriched in heavy peptides in both yeast and hyphae, yellow dots show proteins only enriched in heavy peptides in the yeast form and grey dots indicate proteins that show no heavy peptide enrichment. Proteins detected in the yeast IPs or hyphal IPs but not detected in the respective lysates are listed in Supplementary Dataset S2.

We have previously demonstrated the ability to perform SILAC labelling in *C*. *albicans*^23^. In this study, we used SILAC labelling in conjunction with AP-MS analysis to identify the interacting partners of Cdc14^PD^-Myc. A schematic of the SILAC workflow used in this study is shown in Fig. 2a. The SILAC methodology is designed to distinguish the large number of proteins that will non-specifically bind to the beads during affinity purification, from the proteins specifically attached to the bait (Cdc14^PD^-Myc). This is especially important in the present context because washes were kept to minimum to preserve transient interactions. The bait culture is labelled with heavy isotopes, while a parallel light culture without the bait is unlabelled. Cells from the two cultures are mixed in equal quantities, lysed, and Cdc14^PD^-Myc immunoprecipitated. Proteins bound non-specifically to the beads will be derived in equal quantities from both cultures. Thus, during MS analysis there will be 1:1 heavy to light (H:L) ratio of peptides derived from these proteins. Proteins specifically interacting with the Cdc14^PD^-Myc bait will only originate from the heavy lysate and thus show a H:L ratio greater than 1:1. All SILAC experiments were performed using the strain *CDC14/MET3-Cdc14*^*PD*^*-Myc* as bait in *MET3*-derepressing conditions. This strain was grown in the presence of heavy labelled amino acids, arginine (Arg10) and lysine (Lys8). We previously showed that Arg10 was efficiently incorporated into proteins even in strains that are arginine prototrophs^23^. Cells from the wild type strain (light) were mixed with an equal quantity of Cdc14^PD^ cells (heavy) prior to affinity purification. Mixing of cultures before affinity purification has been shown to provide greater accuracy because it disfavours non-physiological but specific interactions that may occur after lysis^24^. Therefore, we chose this approach to ensure only physiologically stable interactions of Cdc14 were identified. MS analysis was carried out on the IP to determine the H:L ratio of proteins identified (see Fig. 2a). As a further control, we also measured protein abundance using quantitative MS analysis in the combined lysate before Cdc14^PD^ was affinity purified.

The MS output from two biological replicates from yeast cells and two biological replicates from hyphal cells were processed using the MaxQuant and Perseus software suites^25–27^. Proteins enriched in heavy isotopes were identified by the Significance B test based on Benjamini-Hochberg procedure using protein H/L ratio normalised relative to protein intensity and setting false discovery rate to 0.05 as described in Materials and Methods. After removal of proteins that were already enriched in heavy isotopes in the lysate before affinity purification, a set of 126 such proteins were identified (Supplementary Information Table S1 and Supplementary Dataset S1). In this set, 34 proteins were enriched in the IP of both yeast and hyphae, 83 proteins were only enriched in the yeast IP and 9 proteins were only enriched in the hyphal IP. The output from MaxQuant is shown graphically in Fig. 2b as a plot of the signal intensity against H:L ratio after proteins that were enriched in the combined lysate have been removed. In the hyphal IP, proteins significantly enriched in heavy peptides are shown in blue, and proteins enriched in the yeast IP, but not in hyphal IP, are shown in yellow. In the yeast IP, proteins that are also enriched in the hyphal IP are shown in blue, and proteins enriched only in the yeast IP are shown in yellow. In both the yeast and hyphal IPs, proteins detected, but not significantly enriched, are shown in grey. The highlighted proteins are clearly separate from the grey background. The set of 83 proteins whose H:L ratio was significantly elevated only in yeast show a lower level of enrichment than those with a significantly elevated H:L ratio in both yeast and hyphae. Of these proteins, 47 were not even detected in the hyphal IP. For the remaining 35, we tested the possibility that as a group these proteins are enriched in hyphae, but were missed due to lack of statistical power. H:L ratios of proteins significant only in yeast IPs were evenly distributed in hyphae IPs, suggesting this is not the case (Fig. 2b, *p* = 0.93, Wilcox gene set test). In order to provide an independent measure of reproducibility, we plotted the H:L values in each biological replicate of those proteins that were significantly enriched in the MaxQuant-Perseus analysis (Fig. 2c). The correlation between biological replicates from yeast IP was 0.8 (p < 10^−15^) for the yeast replicates, and 0.83 (p < 2 × 10^−6^) for the hyphal replicates, showing a high degree of reproducibility. Note 34 proteins were independently identified in the two yeast biological replicates and the two hyphal biological replicates and are thus identified on the basis of four independent biological replicates.

### Function of Cdc14-interacting proteins

**Figure 3:**
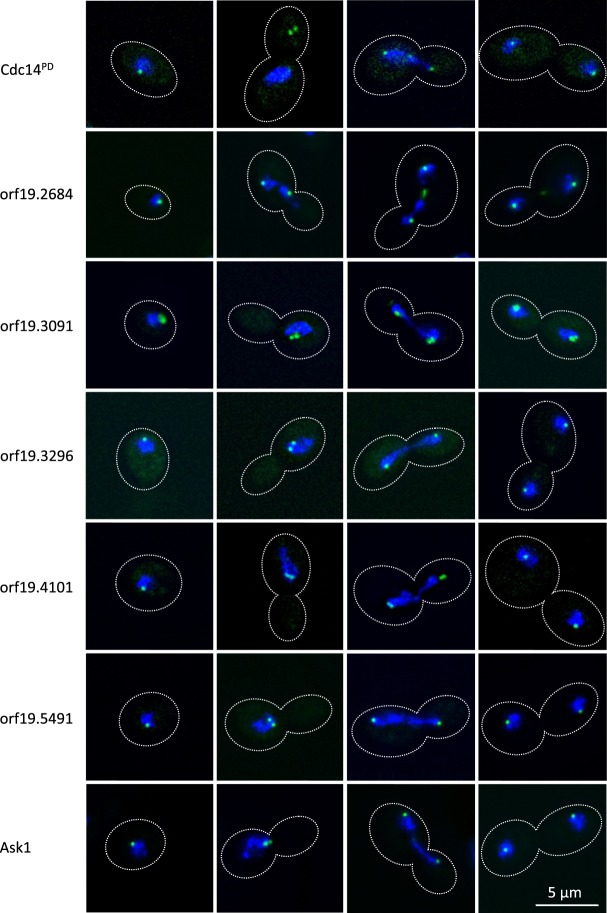
Five ORFs of previously unknown function localise to the SPB. C-terminal fusions of the indicated ORFs to GFP were constructed (Table 1). Overnight cultures of yeast cells were re-innoculated into YEPD yeast-promoting conditions and incubated for 4 hours. Cells were fixed and stained with DAPI. GFP and DAPI fluorescence was imaged using a Delta Vision RT microscope as described in materials and methods. Representative images of cells at different stages of the cell cycle are shown as maximum intensity projections of the green GFP signal and the blue DAPI signal.

Amongst the list of Cdc14 interacting proteins were 14 ORFs of unknown function (Supplementary Information Table S1). We sought to further investigate the functions of these ORFs by generating C-terminal GFP fusions (Table 1) and examining their localisation in yeast (Fig. 3). For comparison we examined the localisation of Ask1-GFP, which was identified as an interacting protein to Cdc14^PD^ in our study. Ask1 is a member of the DAM/DASH complex and a known substrate of Cdc14 in *S*. *cerevisiae*. The localisation studies revealed that five of the fourteen unknown ORFs: ORF19.2684-GFP, ORF9.3091-GFP, ORF19.3296-GFP, ORF19.4101-GFP and ORF19.5491-GFP localised to the Spindle Pole Body l (SPB) similar to the localisation of Ask1-GFP (Fig. 3). That is, in unbudded cells these proteins appeared as a single small dot on the periphery of the nucleus, which then duplicated, migrated to opposite sides of the nucleus and then to opposite poles of separating nuclear material during anaphase. ORF19.2684 was also seen in the mid-section of the mitotic spindle after anaphase, suggesting that a pool also localises to the midzone (Fig. 3). ORF19.3091 showed an additional more diffuse area of localisation next to the fluorescence of the dot presumed to be the SPB.

**Table 1:** Strains used in this study.

Strain	Genotype	Reference
MDL04	*lys2::CmLEU2/lys2::CdHIS1 arg4Δ/arg4Δ leu2Δ/leu2Δ his1Δ/his1Δ ura3Δ::imm434/ura3Δ::imm434 iro1Δ::imm434/iro1Δ::imm434*	Munro lab, University of Aberdeen
BWP17	*ura3::imm434/ura3:imm434 his1::his1G/his1::his1G arg4::hisG/arg4::hisG*	Wilson *et al*., 1999
MET3-Cdc14^PD^-Myc	MDL04 *CDC14/ARG4::MET3-cdc14C275S-MYC::URA3*	Kaneva *et al*.^23^
MET3-Cdc14/Cdc14^PD^-Myc	MDL04 *ARG4::MET3-CDC14/cdc14C275S-MYC::URA3*	This study
Cdc14^PD^-Myc/Mlc1-GFP	MDL04 *CDC14/cdc14C275S-MYC::URA3 MLC1/MLC1-GFP::ARG4*	This study
Cdc14^PD^-GFP	MDL04 *CDC14/cdc14C275S-GFP::ARG4*	This study
Ask1-GFP	BWP17 *ASK1/ASK1-GFP::HIS1*	This study
ORF19.2684-GFP	BWP17 *19*.*2684/19*.*2684-GFP::HIS1*	This study
ORF19.3091-GFP	BWP17 *19*.*3091/19*.*3091-GFP::HIS1*	This study
ORF19.3296-GFP	Bwp17 *19*.*3296/19*.*3296-GFP::HIS1*	This study
ORF19.4101-GFP	BWP17 *19*.*4101/19*.*4101-GFP::HIS1*	This study
ORF19.5491-GFP	BWP17 *19*.*5491/19*.*5491-GFP::HIS1*	This study

We also investigated the localisation of Cdc14^PD^-GFP (Fig. 3). The results show that the localisation was similar to Ask1-GFP and the five ORFs described above, consistent with the idea that Cdc14 is co-localising at the SPB with the ORFs that we have identified as interacting partners of Cdc14. However, there is an interesting anomaly in that we consistently found that when the SPB had duplicated, but before the onset of anaphase, Cdc14^PD^-GFP did not co-localise with the nucleus, but was found in the bud (see Fig. 3). The localisation of Cdc14^PD^ we observed is in contrast to a previous report of Cdc14-GFP localisation, where it was found that Cdc14 only localises to the SPB in early mitosis and to the bud neck after anaphase^14^. In our hands, we were unable to clearly visualise Cdc14-GFP, whereas Cdc14^PD^-GFP clearly localised to the SPB. This may be because Cdc14^PD^ makes a more stable interaction with its substrate thus providing a stronger localisation signal.

To provide further insight into the potential function of these ORFs of unknown function, we queried the InterPro data base to identify domains and motifs that might suggest a potential function^28^. We found that ORF19.7060 contains a XLF double strand break repair domain, which promotes non-homologous end joining (PF09302, IPR015381). An XLF domain is also found in *S*. *cerevisiae* Nej1 known to play a role in double strand break repair, although there is no obvious homology detectable using BLAST to compare Nej1 and ORF19.7060. Finally, we found that ORF19.5491 contains an Afadin/alpha-actinin-binding (IPR021622) domain, which in *S*. *pombe* anchors spindle pole bodies to spindle microtubule, consistent with the observation that this protein does indeed localise to the SPB as described above.

**Figure 4:**
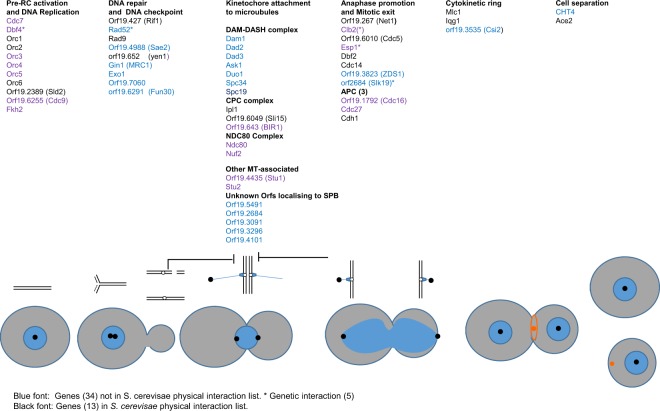
Role of 55 *Ca*Cdc14-interacting proteins in the cell cycle. The cartoon represents the cell and nuclear cycles. Blue circles represent nuclei, black dots SPBs, chromosomes are shown above, replicating early in the cycle and then attached to MTs (blue lines) through kinetochores before sister chromatid separation at anaphase. Thick black lines show DNA damage and spindle checkpoints. After anaphase a contractile actomyosin ring forms (orange ring) which then contracts to a spot (orange) to guide the formation of the primary septum during cytokinesis before cell separation. Proteins also known to physically interact with *Sc*Cdc14 are shown in black font, other proteins are shown in blue font. Where the *S*. *cerevisiae* orthologue is shown in brackets the standard name in CGD is different, but the *S*. *cerevisiae* name is annotated as an alias or homolog in CGD. Protein functions are taken from the annotation in SGD or CGD. ORF19.7060 is included in DNA repair category as it contains and InterPro XLF domain implicated in double strand break repair (see text). CPC: Cargo passenger complex. APC: Anaphase promoting complex.

In the combined set of 126 proteins significantly enriched in one or both IPs, 55 proteins are known, (from research in *S*. *cerevisiae* or *S*. *pombe*) to have a role in either DNA replication and double strand DNA damage repair, chromosome segregation, kinetochore attachment to microtubules, spindle organization, regulation of mitotic exit, cytokinesis, septum formation and licencing of the DNA pre-replication complex; or as shown here to localise to the SPB (Supplementary Dataset S3) and Fig. 4). The 55 proteins include a majority of proteins that were present in both the yeast and hyphal IPs (29/34); a further 3 were only significant in the hyphal IP. Of the 55 proteins shown in Fig. 4, 18 are documented in the *Saccharomyces* Genome Database (SGD) as physical interactors with Cdc14 in *S*. *cerevisiae* (shown in black font in Fig. 4) and a further 5 are documented as genetic interactors (marked with an asterisk in Fig. 4). Gene Ontogeny analysis of the set of 83 yeast-specific Cdc14 interactors in *C*. *albicans* showed a significant enrichment of proteins involved in ergosterol biosynthesis (9 out of 83 proteins (11%) compared to 24 out of 2743 background proteins (0.9%), FDR $\approx$ 0%). These proteins included Ncp1 that encodes NADPH-cytochrome P450 reductase, which partners Erg11, the target of azole antifungals, in sterol 14 alpha-demethylation, and Mcr1 which in *S*. *cerevisiae* has been reported to partner Erg11 in the same reaction^29^.

### Comparative analysis of Cdc14 interacting proteins in *C*. *albicans*, *S*. *pombe* and *S*. *cerevisiae*

We compared the concordance of the set of *Ca*Cdc14 interacting proteins identified in this study with the set of proteins that have been shown to physically interact with *Sc*Cdc14 and *S*.*pombe* Clp1. To do this, we retrieved a non-redundant list of 219 proteins that physically interact with Cdc14 from the *Saccharomyces* Genome Database (SGD). Of the 126 *C*. *albicans* Cdc14 interacting proteins identified in this study, 17 were in this set of *S*. *cerevisiae* Cdc14 interactors or 18.3% of 94 proteins in the *C*. *albicans* set of Cdc14 interactors that had an identifiable *S*. *cerevisiae* homolog. This represents a 5.4-fold enrichment over a random sample (*p* = 8.5 × 10^−10^, hypergeometric test). In *S*. *pombe*, Chen *et al*. identified 138 Clp1/Cdc14 physical interactors^15^. Of these, three have an identifiable homolog in the set of *C*. *albicans* Cdc14 interactors or 5.4% of the 56 genes in the *C*. *albicans* set that have an identifiable *S*.*pombe* homolog (Supplementary Dataset S5). This does not represent a significant enrichment. However, this calculation is compromised because of the low number of *S*. *pombe* homologs listed in Candida Genome Database (CGD). The Chen *et al*. survey of Clp1 interactors in *S*. *pombe* showed enrichment in the similar processes such as chromosomes segregation, cytokinesis, DNA replication and repair^15^. Furthermore, our manual curation identified a number of homologs in both sets that were not listed as homologs in CGD. For example, *S*. *pombe* genes SPCC1223.15c and SPBC32F12.08c are annotated in PomBase as Spc19 and Duo1 respectively, but *CaSPC1*9 and *CaDUO1* are not annotated as *S*. *pombe* homologs in CGD. We identified only two genes, Sli15 and Iqg1 that are targets in all three Cdc14 interaction sets. However, the problematic annotation of *S*. *pombe* homologs in the CGD database means that this is unlikely to be a complete list.

### Enrichment of Cdk1 target sites in the *C*. *albicans* Cdc14-interacting proteins

**Figure 5:**
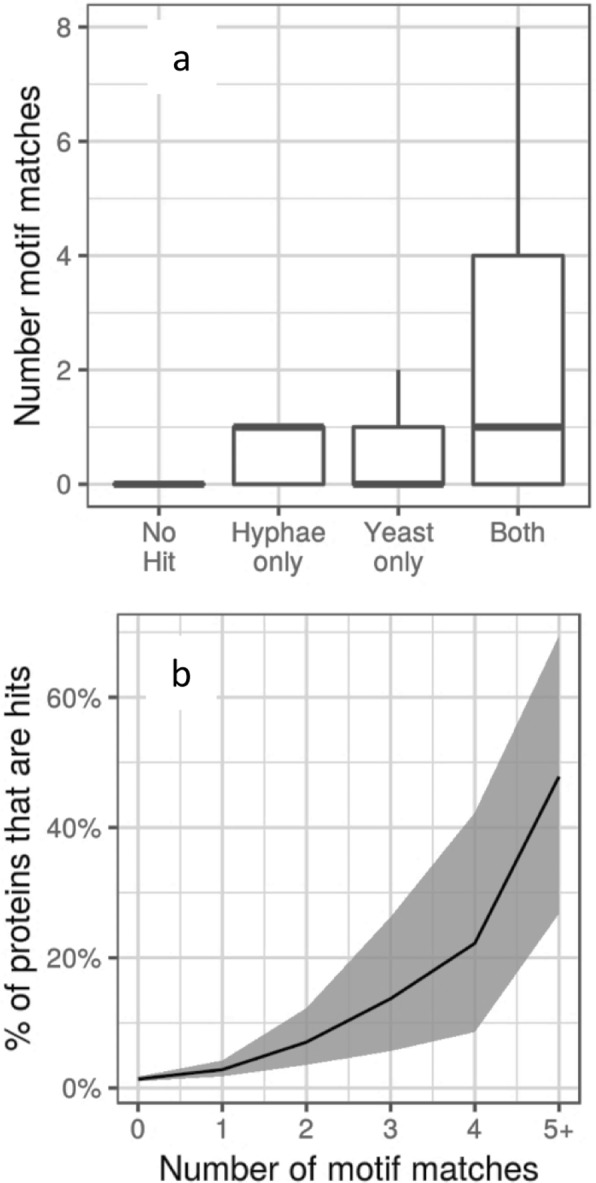
Cdc14 dephosphorylation motifs are enriched in the set of Cdc14 interacting proteins. (**a**) Box plots of the number of consensus Cdc14 dephosphorylation motifs found proteins classified according to whether they were identified in a set of Cdc14-interacting proteins (hits) in hyphae only, yeast only, both yeast and hyphae or not present in the total set of hits. Boxes show the second and third quartiles, the thick horizontal line shows the median, and the vertical line shows the extent of samples within 1.5x of the interquartile range. (**b**) Plots of the percentage of all proteins carrying the indicated number of target motifs that were identified in the set of hits. The shaded area shows the 95% confidence limits.

A structural study has shown that Cdc14 preferentially dephosphorylates the targets of proline-directed kinases, consistent with its role in reversing the phosphorylation by Cdk1^30^. It has further been shown that Cdc14 specifically dephosphorylates serine rather than threonine and has a preference for basic residues at position + 3^31^. In the *C*. *albicans* proteome, 18% of all proteins contain an SPx(R|H|K) motif. However, this motif is present in 44% of both the yeast and hyphal hits, representing a significant enrichment of Cdc14 target motif in proteins we identified as Cdc14-interactors. (Odds Ratio of 8.3, p < 2.2e^−16^ (Fisher exact test) for the yeast list and an Odds Ratio of 9.1, p < 1.1e^−11^ (Fisher exact test) for the hyphal list). A list of these proteins is presented in Supplementary Dataset S4, which shows that 37 of the 58 proteins contain more than one dephosphorylation motif. Two significant trends are evident in this list. First, a higher proportion of Cdc14-interacting proteins that were identified in both yeast hyphae IPs contain a Cdc14 dephosphorylation motif (Fig. 5a). This provides further confidence that such proteins are true interactors with Cdc14 and most likely the interaction is catalytic. Second, as the number of Cdc14 dephosphorylation motifs in a protein increases, there is an increasing likelihood that the protein is present in the combined yeast and hyphal set of hits (Fig. 5b). This is a very significant trend (p < 2.2 × 10^16^ on a logistic regression). There are 23 proteins with five or more Cdc14 dephosphorylation motifs in the *C*. *albicans* proteome, of which 11 (48%) are present in our list of hits. If multiple dephosphorylation sites increases the probability that a protein is a target of Cdc14, then this trend shows that a substantial fraction of all such proteins in the *C*. *albicans* proteome are present in the combined list of interactors.

Recently, a set of peptides that promote Cdc14 substrate binding was identified from screen of a 16-residue library of peptides representing the disordered regions of the *S*. *cerevisiae* proteome^32^. Such peptides contained an RxL motif in a more loosely constrained hydrophobic environment defined as Φ-x-x-ω-P-x-L-x-Φ, where Φ is a large hydrophobic or aromatic side chain and ω is a small hydrophobic side chain. We investigated whether the simple RxL or the extended motif was enriched in the list of proteins we identified, but failed to see any convincing evidence of enrichment (Odds Ratio of 1.65 for the simple motif). We also saw only weak evidence for enrichment in the set of proteins that physically interact with Cdc14 in *S*. *cerevisiae* with RxL giving highest Odds Ratio of 1.9, compared to an Odds Ratio of 4.4 for an enrichment of the S-P-x-(R|H|K) Cdk1 target motif. We note that Kateria *et al*. also found that these motifs were not enriched in *S*. *cerevisiae* proteins that have been experimentally demonstrated to be dephosphorylated by Cdc14^32^. These authors commented that there was considerable variation in the sequence of the peptides recovered in their screen and that genuine interaction motifs are difficult to distinguish from the chance occurrence of similar peptide sequences.

We investigated whether any amino acid motif was enriched in the 126 hits that might be utilized for Cdc14 binding. To do this we used the MEME Motif discovery tool^33^. Amongst the enriched motifs those matching the most sites were rich in polar non-charged amino acids, particularly glutamine. A 12 amino acid motif with a strong preference for Q at each position appeared 51 times in the 126 proteins with an E-value = $9.6\times {10}^{-127}$. In addition, there was enrichment for sequences enriched in threonine (E-value = 5.5 × 10^−32^) and asparagine (E-value = 5.5 × 10^−24^).

## Discussion

Identification of the targets of enzymes such as kinases and phosphatases is a key objective in studying their intracellular role. A common approach is to identify the proteins with which they physically interact by affinity purification and mass spectrometry. We used a substrate-trapping approach combined with SILAC to efficiently discriminate between genuine interactors and proteins which bind non-specifically to the matrix. The application of affinity purification in conjunction with quantitative mass spectrometry using SILAC labelling is a powerful and widely used method to study protein function. This quantitative method readily enables the identification of interacting proteins to be distinguished from the background with high confidence including those with small H:L ratios^24,34–38^. Using this method, we identified 126 Cdc14 interactors that are highly enriched with canonical SPx(R|H|K) motif targeted for dephosphorylation by Cdc14. We identified a set of core interacting partners for Cdc14 in both yeast and hyphae. These partners have clearly identifiable and diverse roles in the cell cycle (Fig. 4) consistent with previous knowledge of Cdc14. However, only 18 of the 55 proteins shown in Fig. 4 are documented to physically interact with Cdc14 in *S*. *cerevisiae* in SGD (shown in black font in Fig. 4). Thus, our study has identified a set of proteins that interacts with Cdc14 in *C*. *albicans*. Within this core set were proteins of previously unknown function, which we showed localised to the Spindle Pole Body and thus are likely to have a role in mitosis. The enrichment of the Cdc14 target motif, the known cell cycle role of 55 of the 126 proteins and the SPB location of five unknown ORFs all provide validation of the screen.

Our study focussed on the interacting partners of Cdc14 and we have not shown that these proteins are dephosphorylated by Cdc14. So, it is possible that interaction is not catalytic, but perhaps the interacting protein is acting as a scaffold. However, the 126 proteins are highly enriched in the SPx(R|H|K) motif that is specific for dephosphorylation by Cdc14. Moreover, we were using a substrate-trapping form of Cdc14 that will thus bind to its substrate through its catalytic specificity. Thus, it is more likely that the interaction is catalytic, but we have not ruled out a non-catalytic role. It is also important to note that in several cases, such as the DAM1/DASH complex and the Origin Recognition Complex, we detected multiple members of the same complex. The presence of multiple members of a protein complex does not indicate that all such proteins are partners of Cdc14, because Cdc14 may target one component of a complex that remains intact during immunoprecipitation. This issue is also relevant to the motif search, because again the motif may be present in proteins which are not Cdc14 substrates, but which are in a complex that is a Cdc14 interactor. However, despite these reservations we have identified diverse functions in the cell cycle in which Cdc14 is involved and provide a database of proteins that physically interact with Cdc14 in *C*. *albicans*.

We identified a total of 43 interacting proteins in hyphae compared to 117 in yeast. Figure 2b shows that there was greater variance in the distribution of H:L ratios in the hyphal IP compared to the yeast IP. This may have made it more difficult to detect proteins that are significant outliers. The reduced abundance of Cdc14 in hyphae compared to yeast, which we noted above, may have contributed to this issue. The majority of the Cdc14-interacting proteins identified in fungi (34/43) were also identified in the yeast IP. Thus, these proteins were identified on the basis of four biological replicates. Moreover, these proteins were more enriched in the canonical Cdc14 dephosphorylation motif compared to proteins only enriched in the yeast IP. Finally, the majority (29/34) of proteins enriched in both the yeast and hyphal IPs, were included in the set of proteins playing identifiable roles in the cell cycle. Taken together, these observations provide powerful evidence that these proteins are genuine Cdc14 interactors.

Although we identified 126 interacting partners in the hyphal and/or yeast IPs, this is unlikely to be the complete list. Indeed, we did not detect either Gin4 or Nap1, which are two proteins that have been shown in low-throughput studies to be substrates of Cdc14^39,40^. Furthermore, dephosphorylation of Sic1 by Cdc14 is a critical step in mediating mitotic exit in *S*. *cerevisiae*. There is no Sic1 homolog in the *C*. *albicans* genome, but a functional homolog, Sol1, has been described, which was also not in the list of Cdc14 interactors identified in this study^41^. An alternative approach to the identification of Cdc14 targets was based on a survey in *S*. *cerevisiae* of peptides showing changes in phosphorylation in a *cdc14-1ts* strain compared to a wild type strain^42^. GO analysis of the hits in this survey again showed enrichment in proteins involved in cell cycle progression. However, in this study the bud site selection marker Bud3, and the condensing complex component Smc4 were shown to be Cdc14 substrates. However, these proteins were also not identified in this study.

## Conclusion

By combining SILAC labelling in conjunction with affinity purification-mass spectrometry using a substrate-trapping mutant we have identified a set of Cdc14-interacting proteins in a way that greatly reduces false positive identification through proteins that non-specifically bind to the affinity matrix. We show that a core set, largely present in both yeast and hyphal IPs, are involved in diverse roles in the cell cycle. While these roles are consistent with the known role of Cdc14 in *S*. *cerevisiae* and *S*. *pombe*, we have greatly expanded the number of potential substrates in *C*. *albicans*. We have identified probable roles for five ORFs of previously unknown function. This dataset provides a secure foundation for further exploration of the role Cdc14 in *C*. *albicans* and thus the mechanisms of chromosome stability in an important human pathogen.

## Materials and Methods

### Strains and growth conditions

Strains are listed in Table 1 and were constructed using methods described previously^22,43^. A list of oligonucleotides used in strain construction is listed in Supplementary Table 2. Generation of *CDC14/cdc14*^*PD*^*-MYC* is described previously^23^. *CDC14/cdc14*^*PD*^*-GFP* was generated by replacing the *MYC* sequence with a *GFP* sequence in *CDC14/cdc14*^*PD*^*-Myc*. The *MET3* promoter cassette was inserted in front of either alleles of *CDC14* in *MET3*-*CDC14/cdc14*^*PD*^*-Myc* as using a pFA-cassette^43^. *MET3-CDC14/cdc14*^*PD*^*-Myc* and *CDC14/MET3-cdc14*^*PD*^*-Myc* clones were identified by Western blot and DNA sequencing. All GFP-tagged strains were also generated using pFA-cassettes^43^.

### Growth media and conditions

Generally, yeast cells were grown in YPED or SD medium at 30 °C, and hyphae were grown in the same medium plus 20% calf serum at 37 °C as previously described^44^. Media containing the heavy isotopes Lys8 (^13^C_6_, ^15^N_2_) and Arg10 (^13^C_6_, ^15^N_2_) is referred to as heavy media, while light media contains light isotopes of all amino acids present in it. Heavy or light *MET3*-inducing and *MET3*-repressing media for SILAC were prepared as described previously^23^. For the purpose of SILAC experiments with yeast, *CDC14/MET3-cdc14*^*PD*^*-MYC* cells were grown overnight in heavy *MET3*-repressing media and then re-inoculated into 0.5 L heavy *MET3*-derepressing media at OD_595_ = 0.25. Cells were grown in heavy media until culture OD_595_ = 0.7 (approx. 4 hours) and then pelleted. For SILAC experiments in hyphae, *CDC14/MET3-cdc14*^*PD*^*-MYC* yeast cells were grown overnight in heavy *MET3*-repressing media, and then they were transferred into 0.1 L pre-warmed (at 37 °C) heavy *MET3*-inducing media plus serum at OD_595_ = 0.4. Cells were allowed to grow as hyphae in six separate flasks for 60, 75, 90, 105, 120 and 135 min. After that, hyphae from all flasks were harvested by centrifugation, mixed together, and processed further as one sample. MDL04 yeast or hyphae were grown in the exact same conditions except that light media was used instead of heavy media.

### Immunoprecipitation

Cell pellets were flash-frozen in liquid nitrogen immediately after harvesting and then thawed on ice. Equal wet weights of pellets from the light and heavy culture were mixed together and re-suspended in equal volume of ice-cold lysis buffer (20 mM HEPES, 150 mM NaCl, EDTA-free protease inhibitors (Roche), 1 mM PMSF, pH 7.4). Cells were broken in a high pressure cell disrupter (Constant Systems Ltd.) at 35 PSI, 4 °C. Lysates were then centrifuged for 20 min at 4 °C to clear the cell debris. EZview^TM^ Red Anti-c-Myc Affinity Gel (Sigma Aldrich) were used to immunoprecipitate Cdc14^PD^ from the lysate. 100 µl of bead slurry were washed 3 times with lysis buffer and then incubated with the cell lysate for 1 hour at 4 °C. The beads were washed once with lysis buffer and antigens were eluted by heating at 95 °C for 5 min. Proteins were separated by SDS-PAGE and digested with trypsin as described previously^23^.

### Mass Spectrometry and data analysis

Sample preparation, mass spectrometry and data processing in MaxQuant v.1.5.2.8^26^ were carried out as described previously^23^. In addition, efficiency of isotope incorporation in the heavy-labelled strain was also assessed as described^23^. Two biological replicate IPs were carried out from yeast and also two biological replicate IPs were done using hyphae. Following LC MS analysis the replicate MS data from each cell form was merged prior to analysis using MaxQuant. MaxQuant provides high resolution identification of isotope-labelled peptide pairs, measures their intensity and identifies the proteins from which they were derived^25,26^. Perseus analyses the output from MaxQuant and uses both the H:L ratio and signal intensity to generate a permutation-based False Discovery Rate (FDR) for the proteins in the IP that are significant outliers^27^. In order to generate this FDR value it is necessary to combine the data from biological replicates in a single MaxQuant-Perseus analysis^25,27^.Peptide ratios were corrected for arginine-to-proline conversion using the Shiny-Pro6 correction method as described previously^23^. Statistical analysis of the data was carried out in Perseus v.1.2.5.6^27^, where proteins enriched in heavy isotopes were identified by the Significance B test based on Benjamini-Hochberg procedure using normalised protein H/L ratio relative to protein intensity and setting false discovery rate to 0.05. All raw MS data files have been deposited to the ProteomeXchange Consortium via PRIDE partner repository with the dataset identifier PXD009581.

### Analysis of high confidence hits

Where available, functional annotations were taken directly from the *Candida* Genome Database (CGD) (http://www.candidagenome.org/). ORFs with no annotated function were first used as the query sequence in protein BLAST searches against the *Saccharomyces* Genome Database (SGD) (https://www.yeastgenome.org) and subsequently for BLAST searches in GenBank. Proteins which still remained with unassigned functions were then used as query sequences for Interpro protein domains (www.ebi.ac.uk/interpro/). Gene Ontogeny (GO) analysis was generated for *C*. *albicans* using the Termfinder tool on the CGD database. A list of Cdc14 physical interactors was downloaded from the *Saccharomyces* Genome Database. The list of *S*. *pombe* interactors was taken from Table S3 of reference^15^.

We determined proteins carrying the Cdk28 phosphorylation site by searching for the regular expression ‘(ST)P.x.(KR)’ using a custom python script and testing for significance of the overlap using the ‘Fisher.test‘ R function (Supplementary Dataset S5).

To test for enrichment of yeast only hits in the hyphae data, we ranked proteins present in the hyphae data by their −log_10_ B significance and used a Wilcox gene set test, as implemented in the WilcoxGST function from the limma R package^45^, to test if proteins with significant H:L ratios in yeast only had higher ranks than would be expected. We used a similar process to test for enrichment of ergosterol related proteins. Enrichment plots in S1 Figure were produced using the ‘barcodeplot‘ function from limma (Supplementary Dataset S5).

We determined motifs enriched in Cdc14 hits using the MEME algorithm. We built a background model using *C*. *albicans* protein sequences that were hits in neither yeast nor hyphae and then used this together with sequences that were hits to search for over-represented motifs of between 5 and 15 amino acids using an Any Number of Repeats (ANR) model. We report the top 3 motifs.

### Microscopy

Samples for imaging were withdrawn from mid-log phase cultures. Cells were fixed in 70% ethanol for 1 min and stained with DAPI for 1 min. Wide field epifluorescence microscopy was carried out using a Delta Vision RT microscope (Applied Precision Instruments, Seattle) using an Olympus 100x UPlanAPo NA 1.35 lens (Olympus Tokyo, Japan). Z-stacks with 0.2 µm interval between each image were acquired with 0.2 second exposure for each image. The images were deconvolved with Softworx^TM^ software. Post-acquisition processing was carried out using the FIJI implementation of NIH Image J^46^. The images shown in Fig. 3 were selected from fields of cells which were in random stages of the cell cycle. The dotted outlines of the cells were drawn around DIC images which were acquired alongside the fluorescence images.

## Supplementary information

Dataset 1

Dataset 2

Dataset 3

Dataset 4

Dataset 5

Proteins that physically interact with the phosphatase Cdc14 in Candida albicans have diverse roles in the cell cycle

## References

[1] Kullberg, B. J. & Filler, S. G. *Candidemia in Candida and Candidiasis* (ed. Calderone, R. A.) 327–340 (ASM Press, Washington DC 2002).

[2] Pfaller MA, Moet GJ, Messer SA, Jones RN, Castanheira M (2011). Candida Bloodstream Infections: Comparison of Species Distributions and Antifungal Resistance Patterns in Community-Onset and Nosocomial Isolates in the SENTRY Antimicrobial Surveillance Program, 2008–2009. Antimicrob. Agents Chemother..

[3] Runke, M. *Skin and mucous infections in Candida and Candidiasis* (ed. Calderone, R.) 307–325 (ASM Press, Washington 2002).

[4] Johnson A (2003). The biology of mating in Candida albicans. Nat Rev Microbiol.

[5] Wertheimer, N. B., Stone, N. & Berman, J. Ploidy dynamics and evolvability in fungi. Philosophical Transactions of the Royal Society B-Biological Sciences **371** (2016). 10.1098/rstb.2015.0461 PMC5095540 28080987

[6] Berman Judith (2016). Ploidy plasticity: a rapid and reversible strategy for adaptation to stress. FEMS Yeast Research.

[7] Gerstein AC, Berman J (2015). Shift and adapt: the costs and benefits of karyotype variations. Curr Opin Microbiol.

[8] Hickman MA, Paulson C, Dudley A, Berman J (2015). Parasexual Ploidy Reduction Drives Population Heterogeneity Through Random and Transient Aneuploidy in Candida albicans. Genetics.

[9] Hickman MA (2016). The ‘obligate diploid’ Candida albicans forms mating-competent haploids. Nature.

[10] Wurzenberger C, Gerlich DW (2011). Phosphatases: providing safe passage through mitotic exit. Nat Rev Mol Cell Biol.

[11] Stegmeier F, Amon A (2004). Closing mitosis: The functions of the Cdc14 phosphatase and its regulation. Annual review of Genetics.

[12] Mocciaro A, Schiebel E (2010). Cdc14: a highly conserved family of phosphatases with non-conserved functions?. J Cell Sci.

[13] Blow JJ, Dutta A (2005). Preventing re-replication of chromosomal DNA. Nat Rev Mol Cell Biol.

[14] Clemente-Blanco A (2006). The Cdc14p phosphatase affects late cell-cycle events and morphogenesis in Candida albicans. J Cell Sci.

[15] Chen JS (2013). Comprehensive Proteomics Analysis Reveals New Substrates and Regulators of the Fission Yeast Clp1/Cdc14 Phosphatase. Molecular & Cellular Proteomics.

[16] Bloom J (2011). Global Analysis of Cdc14 Phosphatase Reveals Diverse Roles in Mitotic Processes. J Biol Chem.

[17] Ong SE (2006). & Mann,M. A practical recipe for stable isotope labeling by amino acids in cell culture (SILAC). Nature Protocols.

[18] Gruhler A (2005). Quantitative phosphoproteomics applied to the yeast pheromone signaling pathway. Molecular & Cellular Proteomics.

[19] Jiang H, English AM (2002). Quantitative analysis of the yeast proteome by incorporation of isotopically labeled leucine. Journal of Proteome Research.

[20] Ong SE (2002). Stable isotope labeling by amino acids in cell culture, SILAC, as a simple and accurate approach to expression proteomics. Molecular & Cellular. Proteomics.

[21] Wolfe BA, McDonald WH, Yates JR, Gould KL (2006). Phospho-regulation of the Cdc14/Clp1 phosphatase delays late mitotic events in S-pombe. Developmental Cell.

[22] Care RA, Trevethick J, Binley KM, Sudbery PE (1999). The MET3 promoter: a new tool for Candida albicans molecular genetics. Mol Microbiol.

[23] Kaneva, I. N., Longworth, J., Sudbery, P. E. & Dickman, M. J. *Quantitative Proteomic Analysis in Candida albicans Using SILAC-Based Mass Spectrometry*. Proteomics1700278-1700n/a (2018). 10.1002/pmic.201700278 29280593

[24] Hildebrandt A (2017). Interaction profiling of RNA-binding ubiquitin ligases reveals a link between posttranscriptional regulation and the ubiquitin system. Scientific Reports.

[25] Cox J (2009). A practical guide to the MaxQuant computational platform for SILAC-based quantitative proteomics. Nature Protocols.

[26] Cox J, Mann M (2008). MaxQuant enables high peptide identification rates, individualized p.p.b.-range mass accuracies and proteome-wide protein quantification. Nature Biotechnology.

[27] Tyanova S (2016). The Perseus computational platform for comprehensive analysis of (prote)omics data. Nature Methods.

[28] Jones P (2014). InterProScan 5: genome-scale protein function classification. Bioinformatics.

[29] Lamb DC, Kelly DE, Manning NJ, Kaderbhai MA, Kelly SL (1999). Biodiversity of the P450 catalytic cycle: yeast cytochrome b(5)/NADH cytochrome b(5) reductase complex efficiently drives the entire sterol 14-demethylation (CYP51) reaction. FEBS Letters.

[30] Gray CH, Good VM, Tonks NK, Barford D (2003). The structure of the cell cycle protein Cdc14 reveals a proline-directed protein phosphatase. EMBO J..

[31] Bremmer SC (2012). Cdc14 Phosphatases Preferentially Dephosphorylate a Subset of Cyclin-dependent kinase (Cdk) Sites Containing Phosphoserine. J Biol Chem.

[32] Kataria M (2018). A PxL motif promotes timely cell cycle substrate dephosphorylation by the Cdc14 phosphatase. Nature Structural &. Molecular Biology.

[33] Bailey T, Elkan C (1994). Fitting a mixture model by expectation maximization to discover motifs in biopolymers. Proc Int Conf Intell Syst Mol Biol..

[34] Sadewasser A (2017). Quantitative Proteomic Approach Identifies Vpr Binding Protein as Novel Host Factor Supporting Influenza A Virus Infections in Human Cells. Mol Cell Proteomics.

[35] Asselin-Mullen P (2017). Protein interaction network of alternatively spliced NudCD1 isoforms. Scientific Reports.

[36] Kos-Braun IC, Jung I, Koí M (2017). Tor1 and CK2 kinases control a switch between alternative ribosome biogenesis pathways in a growth-dependent manner. Plos Biology.

[37] Greseth MD, Carter DC, Terhune SS, Traktman P (2017). Proteomic Screen for Cellular Targets of the Vaccinia Virus F10 Protein Kinase Reveals that Phosphorylation of mDia Regulates Stress Fiber Formation. Mol Cell Proteomics.

[38] Singh KD (2017). Differential regulation of germ line apoptosis and germ cell differentiation by CPEB family members in C. elegans. PLoS ONE.

[39] Huang, Z. X. *et al*. Phosphoregulation of Nap1 Plays a Role in Septin Ring Dynamics and Morphogenesis in Candida albicans. *Mbio* **5**, (2014). 10.1128/mBio.00915-13 PMC3950511 24496790

[40] Yong JYA, Wang YM, Wang Y (2016). The Nim1 kinase Gin4 has distinct domains crucial for septin assembly, phospholipid binding and mitotic exit. J Cell Sci.

[41] Atir-Lande A, Gildor T, Kornitzer D (2005). Role for the SCFCDC4 ubiquitin ligase in Candida albicans morphogenesis. Mol Biol Cell.

[42] Kao L (2014). Global Analysis of Cdc14 Dephosphorylation Sites Reveals Essential Regulatory Role in Mitosis and Cytokinesis. Molecular & Cellular Proteomics.

[43] Gola S, Martin R, Walther A, Dunkler A, Wendland J (2003). New modules for PCR-based gene targeting in Candida albicans: rapid and efficient gene targeting using 100 bp of flanking homology region. Yeast.

[44] Sudbery PE (2001). The germ tubes of Candida albicans hyphae and pseudohyphae show different patterns of septin ring localisation. Mol Microbiol.

[45] Michaud, J. *et al*. Integrative analysis of RUNX1 downstream pathways and target genes. *Bmc Genomics* **9**, (2008). 10.1186/1471-2164-9-363 PMC2529319 18671852

[46] Schindelin J (2012). Fiji: an open-source platform for biological-image analysis. Nature Methods.

