# Supplementary material for: Proteins that physically interact with the phosphatase Cdc14 in Candida albicans have diverse roles in the cell cycle
Source: Sci Rep. 2019 Apr 18;9:6258. doi: 10.1038/s41598-019-42530-1 (PMC6472416; doi:10.1038/s41598-019-42530-1)
Supplement: Supplementary file 5 — Dataset 5 [file 41598_2019_42530_MOESM5_ESM.zip › Zip/outputs/ANalysis_of_cdc14_overlaps.html]

Overlap of Cdc14 interactors


Code 

- Show All Code
- Hide All Code

# Overlap of Cdc14 interactors

This notebook explores the overlap between cdc14 interactors found in Candida (Ca) by Iliyana and those from the literature for Pombe (Sp) and cerevisiae (Sc).

First we must read in the annotations and mangle them into the correct format

```
library(tidyr)

#setwd("C:/Users/IanSudbery/Google Drive/Projects/pete/")
ca_annotations <- read.delim("C_albicans_SC5314_A19_current_chromosomal_feature.tab", 
                                    comment.char = "!",
                                    header=FALSE,
                                    col.names=c("fname",
                                                "gene_name",
                                                "aliases",
                                                "fType",
                                                "chr",
                                                "start",
                                                "end",
                                                "strand",
                                                "CGDID",
                                                "secondary_CGDID",
                                                "description",
                                                "data",
                                                "genome_version",
                                                "blank1",
                                                "blank2",
                                                "date",
                                                "standard",
                                                "sc_homologue"))
#Each gene has two rows for the A and B allele. Only keep A allele
ca_annotations <- ca_annotations[grepl(pattern = "_A$", ca_annotations$fname),]

#Split concatenated aliases onto different rows
ca_annotations <- separate_rows(ca_annotations, aliases, sep="\\|")

#subset to orf numbers
ca_annotations <- ca_annotations[grepl("orf19.[[:digit:]]+", ca_annotations$aliases),]

head(ca_annotations[ca_annotations$sc_homologue != "",c("fname","aliases","gene_name", "sc_homologue")])
```

```
##          fname     aliases gene_name sc_homologue
## 55 C1_00060W_A  orf19.6109      TUP1         TUP1
## 58 C1_00060W_A orf19.13528      TUP1         TUP1
## 66 C1_00070W_A  orf19.6105       MVD         MVD1
## 69 C1_00070W_A orf19.13524       MVD         MVD1
## 78 C1_00080C_A  orf19.6102      RCA1         CST6
## 81 C1_00080C_A orf19.13521      RCA1         CST6
```

```
ca_hits <- read.delim("ca_interactors.txt")
sc_hits <- read.delim("sc_interactors.txt", comment.char = "!")
sp_hits <- read.delim("sp_interactors.txt")
sp_physical <- read.delim("sp_physical.txt")
ca_hits <- separate_rows(ca_hits, Protein_IDs)
ca_hits <- subset(ca_hits, Gene !="CDC14")
sc_hits <- subset(sc_hits, Gene_Name != "CDC14")
sp_hits <- subset(sp_hits, Protein != "Clp1")
sp_physical <- subset(sp_physical, Gene != "clp1")
```

# Candida and cerevisae overlap

How many of the Ca hits have Sc homologs?

```
table(ca_annotations$sc_homologue[ca_annotations$aliases %in% ca_hits$Protein_IDs] != "")
```

```
## 
## FALSE  TRUE 
##    33    93
```

That is, 93 of the 126 orfids (126 genes) have Sc homologs according to CGD.

How many of the Sc hits have a Ca homolog?

```
table(sc_hits$Gene_Name %in% ca_annotations$sc_homologue)
```

```
## 
## FALSE  TRUE 
##    60   159
```

So 159 of the 219 Sc hits have a Ca homolog according to CGD.

What % of the Ca hits are also in the Sc\_hits list?

```
library(scales)
sc_hits$ca_homologue <- ca_annotations$fname[match(sc_hits$Gene_Name, ca_annotations$sc_homologue)]
ca_hits$fname <- ca_annotations$fname[match(ca_hits$Protein_IDs, ca_annotations$aliases)]

print(table(unique(ca_hits$fname) %in% sc_hits$ca_homologue))
```

```
## 
## FALSE  TRUE 
##   109    17
```

```
sc_hits_with_hom = length(unique(na.exclude(sc_hits$ca_homologue)))
ca_genes_with_sc = length(unique(ca_annotations$fname[ca_annotations$sc_homologue != ""]))
ca_hits_with_sc_hit = sum(unique(ca_hits$fname) %in% sc_hits$ca_homologue)
ca_hits_with_sc_hom = sum(ca_annotations$sc_homologue[ca_annotations$aliases %in% ca_hits$Protein_IDs] != "")

#fraction of ca_hits that are also Sc hits
f_ca_hits_with_sc = ca_hits_with_sc_hit/ca_hits_with_sc_hom

#fraction of genes with Sc homolouges that are sc_hits
f_sc_hits_with_ca_homs = sc_hits_with_hom/ca_genes_with_sc

#enrichment of ca hits which are also sc hits
enrichment_sc = f_ca_hits_with_sc/f_sc_hits_with_ca_homs

#p value on the enrichment
pvalue = phyper(ca_hits_with_sc_hit, sc_hits_with_hom, ca_genes_with_sc - sc_hits_with_hom, ca_hits_with_sc_hom, lower.tail = F)
```

So 17 of the 126 Candida hits were also found in Sc, or 18.3% of genes that have an annotated Sc homologue. Since 159 of the 4012 candida genes with a Sc homologue are hits in Sc, this is a 5.4-fold enrichment, which is significant by hypergeometric test with a p-value of 8.5e-10.

These 17 genes are:

```
subset(ca_hits[,-4], fname %in% sc_hits$ca_homologue)
```

```
##      Gene Yeast Hyphae Protein_IDs       fname
## 3    DBF2     +         orf19.1223 C2_06670C_A
## 18   CDH1     +      +  orf19.2084 C2_00450C_A
## 21   SLD2     +         orf19.2389 CR_03340C_A
## 32   ORC1     +      +  orf19.3000 C1_03070C_A
## 39   ORC6     +         orf19.3289 C1_01000C_A
## 45   IPL1            +  orf19.3474 C6_02320C_A
## 47   MCR1     +         orf19.3507 C6_02040W_A
## 58  CDC28     +         orf19.3856 CR_06050W_A
## 68   RIF1     +      +   orf19.427 C1_05380C_A
## 69   RAD9     +      +  orf19.4275 C5_02610C_A
## 82   ORC2     +      +  orf19.5358 C2_10760C_A
## 91   CDC5     +      +  orf19.6010 C1_00950C_A
## 95  SLI15     +      +  orf19.6049 C1_00570C_A
## 96   ACE2     +      +  orf19.6124 CR_07440W_A
## 108  YEN1     +      +   orf19.652 CR_05010W_A
## 109  IQG1     +         orf19.6536 C7_01840W_A
## 124  CSM1     +         orf19.7663 CR_10740W_A
```

# Candida and pombe overlap

I downloaded a list of Pombe/Candida homologues from the CGD from this address: http://www.candidagenome.org/download/homology/orthologs/C\_albicans\_SC5314\_S\_pombe\_by\_inparanoid/C\_albicans\_SC5314\_S\_pombe\_orthologs.txt

I will use these to do the comparison with the none-coloured pombe hits in the sheet sent by pete.

```
pombe_homologs <- read.delim("ca_sp_homologs.txt", comment.char = '#',
                             header=F,
                             col.names=c("ca_fname",
                                         "ca_gene_name",
                                         "ca_cgdid",
                                         "sp_fname",
                                         "sp_gene_name",
                                         "sp_dbname"))
```

Its worth noting that only 2603 genes have pombe homologs by this method.

```
# Candida hits with Pombe homologs
ca_hits_sp_hom <- sum(ca_hits$fname %in% pombe_homologs$ca_fname)

# pombe hits with candida homolog
sp_hits_ca_hom <- sum(sp_hits$ORF %in% pombe_homologs$sp_fname)

# Annotate pombe hits with candida homologs
sp_hits$ca_homolog <- pombe_homologs$ca_fname[match(sp_hits$ORF, pombe_homologs$sp_fname)]

# Number of Ca hits that are also Pombe hits
ca_hits_sp_hit <- sum(ca_hits$fname %in% sp_hits$ca_homolog)

# Fraction of ca genes with pombe homologs which are sp_hits
f_sp_hits_with_ca_homs <- sp_hits_ca_hom/(dim(pombe_homologs)[1])

# Fracation of ca hits with homologs that are also sp hits
f_ca_hits_with_sp_hit <- ca_hits_sp_hit/ca_hits_sp_hom

enrichment_sp = f_ca_hits_with_sp_hit/f_sp_hits_with_ca_homs

pvalue_sp <- phyper(ca_hits_sp_hit, sp_hits_ca_hom, dim(pombe_homologs)[1] - sp_hits_ca_hom, ca_hits_sp_hom, lower.tail=F)
```

55 Of the candida hits have an annotated pombe ortholog and 71 of pombe hits have a candida ortholog. Of those 55 candida hits, 3 were also hits in pombe, or 5.45%. Given that 71 of 2603 homologs are pombe hits, this equates to 2 fold enrichment and gives a p-value of 0.061.

Thus we conclude that there is no significant overlap between pombe and candida hits.

Those 3 genes are:

```
subset(ca_hits[,-4], fname %in% sp_hits$ca_homolog)
```

```
##     Gene Yeast Hyphae  Protein_IDs       fname
## 24  MLC1     +        orf19.2416.1 CR_03090C_A
## 109 IQG1     +          orf19.6536 C7_01840W_A
## 113 STU2     +          orf19.6610 CR_09520C_A
```

## Pombe hits from pombase

Pombase contains a slightly different set of interactors. It is not clear what this list is. It contains 169 interactors of Clp1, quoting BioGRID as the source. However, bioGRID lists 185 physical interactors of Clp1 (but won’t let you download them independently of the genetic interactions). If you ask pombase to download the list of interactors, you get a list of 6994 genes, which is clearly incorrect. This list has been cut-and-paste from the pombase website. With that caveat:

```
# Annotate pombe hits with candida homologs
sp_physical$ca_homolog <- pombe_homologs$ca_fname[match(tolower(sp_physical$Gene), tolower(pombe_homologs$ca_gene_name))]

# Number of Ca hits that are also Pombe hits
ca_hits_sp_hit2 <- sum(ca_hits$fname %in% sp_physical$ca_homolog)
```

Of the 35 hits from pombase that have a Candida homolog, 0 are also hits in Candida. In fact, it looks to me like this list is enriched in genes that do not have a Candida ortholog.

# Three way comparison

```
library(gplots)
```

```
## 
## Attaching package: 'gplots'
```

```
## The following object is masked from 'package:stats':
## 
##     lowess
```

```
ca_both_homs <- ca_annotations
ca_both_homs$pombe_homolog <- pombe_homologs$sp_fname[match(ca_both_homs$fname, pombe_homologs$ca_fname)]
ca_both_homs <- subset(ca_both_homs, !is.na(sc_homologue) & !is.na(pombe_homolog))

ca_hits_both_homs <- ca_hits$fname[ca_hits$fname %in% ca_both_homs$fname]
sp_hits_both_homs <- sp_hits$ca_homolog[sp_hits$ORF %in% ca_both_homs$pombe_homolog]
sc_hits_both_homs <- sc_hits$ca_homologue[sc_hits$Gene_Name %in% ca_both_homs$sc_homologue]
venn(list(C.albicans=ca_hits_both_homs,
          S.cerevisiae=sc_hits_both_homs,
          S.pombe=sp_hits_both_homs),
    universe = unique(ca_both_homs$fname),
    simplify = FALSE)
```

```
## Warning in drawVennDiagram(data = counts, small = small, showSetLogicLabel = showSetLogicLabel, : Not shown: 000 contains 2356
```

These numbers are smaller than the above because the background set here is the 2547 genes that have homologs in all three species and, for example, 8 of the genes that are found in both Candida and cerevisiae do not have an annotated ortholog in pombe.
