# Supplementary material for: Proteins that physically interact with the phosphatase Cdc14 in Candida albicans have diverse roles in the cell cycle
Source: Sci Rep. 2019 Apr 18;9:6258. doi: 10.1038/s41598-019-42530-1 (PMC6472416; doi:10.1038/s41598-019-42530-1)
Supplement: Supplementary file 5 — Dataset 5 [file 41598_2019_42530_MOESM5_ESM.zip › Zip/outputs/Cdc28_site_enrichment.html]

Cdc28\_site\_enrichment


# Association of Cdc28 binding motifs and Cdc14 MS binding hits¶

In this notebook we will examine if hits from Cdc14 mass spec are enriched in binding site for Cdc28. Data for the hits is provided in an excel sheet with hits for yeast and hyphae. We will download protein sequences as search through them for matches to the Cdc28 binding consensus. Really we should be doing this using a Cdc28 PWM and proper motif matching, but in the absence of such a PWM, we will use consensus sequences provided by Pete : S/T-P-X-K/R or P-X-S/T-X-(K/R){2-5}.

## Preparing the data¶

First we download the Candida protein sequences....

In [1]:

```
! wget http://www.candidagenome.org/download/sequence/C_albicans_SC5314/Assembly22/current/C_albicans_SC5314_A22_current_orf_trans_all.fasta.gz
```

```
--2018-05-10 11:07:47--  http://www.candidagenome.org/download/sequence/C_albicans_SC5314/Assembly22/current/C_albicans_SC5314_A22_current_orf_trans_all.fasta.gz
Resolving www.candidagenome.org (www.candidagenome.org)... 171.67.194.231
Connecting to www.candidagenome.org (www.candidagenome.org)|171.67.194.231|:80... connected.
HTTP request sent, awaiting response... 200 OK
Length: 4919400 (4.7M) [application/x-gzip]
Saving to: ‘C_albicans_SC5314_A22_current_orf_trans_all.fasta.gz.2’

C_albicans_SC5314_A 100%[===================>]   4.69M  1.50MB/s    in 3.8s    

2018-05-10 11:07:51 (1.22 MB/s) - ‘C_albicans_SC5314_A22_current_orf_trans_all.fasta.gz.2’ saved [4919400/4919400]
```

Now we look for motif in each of the protein sequences.

In [2]:

```
from CGAT import IOTools, FastaIterator
import re
import pandas

regex = re.compile("[ST]P.[KR]")
hits_table=dict()
for protein in FastaIterator.iterate(
    IOTools.openFile("C_albicans_SC5314_A22_current_orf_trans_all.fasta.gz")):

    
    fname = protein.title.split(" ")[0][:-2]
    hits_table[fname] = len(regex.findall(protein.sequence))
    
hits_table = pandas.Series(hits_table)
hits_table = hits_table.reset_index()
hits_table.columns = ["fname","nMotifs"]
hits_table.head()
```

Out[2]:

|  | fname | nMotifs |
| --- | --- | --- |
| 0 | C1\_00010W | 0 |
| 1 | C1\_00020C | 0 |
| 2 | C1\_00030C | 0 |
| 3 | C1\_00040W | 0 |
| 4 | C1\_00050C | 0 |

Now to get hold of the excel table:

In [3]:

```
ms_hits = pandas.read_excel("CA_hits_annotatedb.xlsx")
ms_hits.fname = ms_hits.fname.apply(lambda x: x[:-2])
ms_hits.head()
```

Out[3]:

|  | Gene | Yeast | Hyphae | Description | Protein\_IDs | fname | CGDID |
| --- | --- | --- | --- | --- | --- | --- | --- |
| 0 | HAM1 | NaN | + | Putative deoxyribonucleoside triphosphate pyro... | orf19.1108 | C5\_03860W | CAL0000174774 |
| 1 | MSB1 | NaN | + | Putative regulator of transcription; expressio... | orf19.1133 | C1\_03710C | CAL0000199709 |
| 2 | DBF2 | + | NaN | Essential serine/threonine protein kinase invo... | orf19.1223 | C2\_06670C | CAL0000197161 |
| 3 | FCY21 | + | NaN | High affinity, high capacity, hypoxanthine-ade... | orf19.1357 | C2\_09950W | CAL0000195436 |
| 4 | DUO1 | + | + | Subunit of the Dam1 (DASH) complex, which acts... | orf19.1428 | C4\_04220W | CAL0000184353 |

Now we combine these two datasets to create a single table containing the number of motif hits for each protein and whether it was a hit in yeast, hyphae or either yeast or hyphae.

In [4]:

```
hits_table = hits_table.merge(ms_hits[["fname","Yeast", "Hyphae"]], on="fname", how='left')
hits_table.Yeast = hits_table.Yeast == "+"
hits_table.Hyphae = hits_table.Hyphae == "+"
hits_table["MS Hit"] = (hits_table.Yeast) | (hits_table.Hyphae)
hits_table.head()
```

Out[4]:

|  | fname | nMotifs | Yeast | Hyphae | MS Hit |
| --- | --- | --- | --- | --- | --- |
| 0 | C1\_00010W | 0 | False | False | False |
| 1 | C1\_00020C | 0 | False | False | False |
| 2 | C1\_00030C | 0 | False | False | False |
| 3 | C1\_00040W | 0 | False | False | False |
| 4 | C1\_00050C | 0 | False | False | False |

## Testing for association with combined yeast and hyphae hits¶

We are now ready to look at the association between the two.

In [5]:

```
pandas.crosstab(hits_table.nMotifs>0, hits_table["MS Hit"])
```

Out[5]:

| MS Hit | False | True |
| --- | --- | --- |
| nMotifs |  |  |
| False | 4678 | 61 |
| True | 1422 | 65 |

In other words 65/(61+65) = 52% of the mass spec hits have at least one Cdc28 motif, while 1422/(1422+4778) = 23% of proteins that are not a mass spec hit have at least one Cdc28 motif. This is approximately a 2.2 fold enrichment on expectation. To test if this is significant we can do a fisher's exact test:

In [6]:

```
%load_ext rpy2.ipython
```

In [7]:

```
%%R -i hits_table

fisher.test(hits_table$nMotif > 0, hits_table$MS.Hit)
```

```
	Fisher's Exact Test for Count Data

data:  hits_table$nMotif > 0 and hits_table$MS.Hit
p-value = 1.089e-11
alternative hypothesis: true odds ratio is not equal to 1
95 percent confidence interval:
 2.419739 5.081168
sample estimates:
odds ratio 
  3.504469
```

Thus we can conclude that this enrichment is very statistically significant, with an odds ration of 3.5 and a p-value of $1 \times 10^{-11}$.

Maybe we should go one step further - many Cdc28 targets have multiple binding sites. Lets have a look at the association of proteins that have more 2 or more Cdc28 targets and the hits from the MS.

In [8]:

```
pandas.crosstab(hits_table.nMotifs>1, hits_table["MS Hit"])
```

Out[8]:

| MS Hit | False | True |
| --- | --- | --- |
| nMotifs |  |  |
| False | 5710 | 89 |
| True | 390 | 37 |

Thus 29% of the MS hits have 2 or more Cdc28 sites, while only 6% of non-MS hits have 2 or more Cdc28 sites, a 4.3 fold enrichment.

In [9]:

```
%%R

fisher.test(hits_table$nMotif > 1, hits_table$MS.Hit)
```

```
	Fisher's Exact Test for Count Data

data:  hits_table$nMotif > 1 and hits_table$MS.Hit
p-value = 7.054e-15
alternative hypothesis: true odds ratio is not equal to 1
95 percent confidence interval:
 3.973331 9.158640
sample estimates:
odds ratio 
  6.083147
```

This is of course even more significant, with an odds ratio greater than 6.

## Yeast hits¶

We can repeat the same analysis, but only considering hits in the yeast.

In [10]:

```
pandas.crosstab(hits_table.nMotifs>0, hits_table["Yeast"])
```

Out[10]:

| Yeast | False | True |
| --- | --- | --- |
| nMotifs |  |  |
| False | 4682 | 57 |
| True | 1427 | 60 |

Thus 51% of hits the yeast have Cdc28 motifs, and 23% overall, this looks very similar to the overall figures.

In [11]:

```
hits_table.dtypes
```

Out[11]:

```
fname      object
nMotifs     int64
Yeast        bool
Hyphae       bool
MS Hit       bool
dtype: object
```

In [12]:

```
%%R -i hits_table
fisher.test(hits_table$nMotif > 0, hits_table$Yeast)
```

```
	Fisher's Exact Test for Count Data

data:  hits_table$nMotif > 0 and hits_table$Yeast
p-value = 9.623e-11
alternative hypothesis: true odds ratio is not equal to 1
95 percent confidence interval:
 2.350418 5.077613
sample estimates:
odds ratio 
  3.452758
```

Overall the comparison for yeast looks very similar to that for yeast or hyphae.

## Mass spec hits in Hyphae¶

Now we turn to mass spec hits that were found in the hyphae.

In [13]:

```
pandas.crosstab(hits_table.nMotifs>0, hits_table["Hyphae"])
```

Out[13]:

| Hyphae | False | True |
| --- | --- | --- |
| nMotifs |  |  |
| False | 4723 | 16 |
| True | 1461 | 26 |

Thus 62% of mass spec hits from the hyphae have the Cdc28 motif, which is an enrichment of 2.7 fold.

In [14]:

```
%%R

fisher.test(hits_table$nMotif > 0, hits_table$Hyphae)
```

```
	Fisher's Exact Test for Count Data

data:  hits_table$nMotif > 0 and hits_table$Hyphae
p-value = 1.568e-07
alternative hypothesis: true odds ratio is not equal to 1
95 percent confidence interval:
  2.704324 10.509158
sample estimates:
odds ratio 
  5.251735
```

The p-value here is a little less significant due to the smaller numbers, but $1 \times 10^{-7}$ is still very significant in anyones books. The Odds ratio of 5.2 is also higher than that for the yeast hits.

We can also look at the enrichment of proteins with two Cdc28 motifs in the hyphae hits

In [15]:

```
pandas.crosstab(hits_table.nMotifs>1, hits_table["Hyphae"])
```

Out[15]:

| Hyphae | False | True |
| --- | --- | --- |
| nMotifs |  |  |
| False | 5778 | 21 |
| True | 406 | 21 |

Exactly half of the hyphae hits have two or more Cdc28 motifs. This compares to only 6.6% of those that are not hyphae hits, a 7.6fold enrichment.

In [16]:

```
%%R

fisher.test(hits_table$nMotif > 1, hits_table$Hyphae)
```

```
	Fisher's Exact Test for Count Data

data:  hits_table$nMotif > 1 and hits_table$Hyphae
p-value = 3.169e-14
alternative hypothesis: true odds ratio is not equal to 1
95 percent confidence interval:
  7.318687 27.628215
sample estimates:
odds ratio 
  14.22122
```

This is the most significant result yet, with a p-value of $3.2 \times 10^{-14}$ and an odds ratio of over 14.

## Conclusions¶

Overall the MS hits are enriched for proteins that carry a perfect match to the Cdc28 binding consensus, with a 2.3 fold enrichment, an odds ratio of 3.5 and a p-value of $1\times10^{-11}$. The enrichment is even stronger for proteins that carry two seperate matches to the motif (4.3X, OR 6, p-value $1\times10^{15}$). These results are similar in yeast, but are even stronger in hyphae, with there being a 7.3 fold enrichment of hyphae hits with 2 seperate Cdc28 binding motifs (OR 14, $p=3.2\times10^{-14}$).
