# Supplementary material for: Proteins that physically interact with the phosphatase Cdc14 in Candida albicans have diverse roles in the cell cycle
Source: Sci Rep. 2019 Apr 18;9:6258. doi: 10.1038/s41598-019-42530-1 (PMC6472416; doi:10.1038/s41598-019-42530-1)
Supplement: Supplementary file 5 — Dataset 5 [file 41598_2019_42530_MOESM5_ESM.zip › Zip/outputs/hits_analysis.html]

What is the status of borderline hits?


# What is the status of borderline hits?

In this notebook we will investigate the relationship between the Cdc14 MS results in yeast and hyphae. The questions we will address are:

- What is the relationship between the hits found in hyphae and those found in yeast? Can we say that the hits in hyphae correspond to the strongest hits in yeast.
- Do the hits that are found in yeast and not in hyphae tend to have a strong, but sub threshold, signal in hyphae.
- Many of the yeast only hits are yeast only because they are excluded from the hyphae results due to their ratio in the non-IPed lysate. Do these hits tend to have an stronger-than-average, but sub-threshold signal in the yeast lysate, particularly those in the ergosterol pathway.

First obtain the data, and manipulate it into a form convienient for further processing:

```
library(dplyr)
```

```
## 
## Attaching package: 'dplyr'
```

```
## The following objects are masked from 'package:stats':
## 
##     filter, lag
```

```
## The following objects are masked from 'package:base':
## 
##     intersect, setdiff, setequal, union
```

```
library(tidyr)
yeast_IP = read.delim("yeast_ms.tsv", stringsAsFactors = FALSE)
hyphae_IP = read.delim("hyphae_ms.tsv", stringsAsFactors = FALSE)
yeast_lysate = read.delim("yeast_lysate.tsv", stringsAsFactors = FALSE)
hyphae_lysate = read.delim("hyphae_lysate.tsv", stringsAsFactors = FALSE)

# for each table we want the protein id, the intensity, the ratio, the B value
# and whether it passes a 5% FDR threshold.

results <- bind_rows(yeast.IP = yeast_IP, 
                     hyphae.IP = hyphae_IP, 
                     yeast.lysate = yeast_lysate, 
                     hyphae.lysate = hyphae_lysate, 
                     .id = "sample_name") %>%
           separate(sample_name, 
                    into=c("condition", "fraction"),
                    remove=FALSE) %>%
           select(sample_name,
                  condition,
                  fraction,
                  H.L.ratio,
                  Intensity,
                  B.significant.FDR.0.05,
                  B.significant.FDR.0.05.1,
                  Protein.IDs)%>%
           rename(significant = B.significant.FDR.0.05,
                  B.value = B.significant.FDR.0.05.1)

per_gene_significance = select(results,
                               Protein.IDs,
                               sample_name,
                               significant) %>%
                        mutate(sample_name = paste0("sig.", sample_name)) %>%
                        spread(key = sample_name,
                               value = significant,
                               fill="Not Present")

results = merge(results, per_gene_significance)
```

Now are ready to look at the classic plots for MS. First we will identify the hits on the plots

```
library(ggplot2)
ggplot(results) +
  aes(H.L.ratio, Intensity, color=significant, alpha=significant) +
  facet_grid(fraction ~ condition) +
  geom_vline(xintercept = 1, lty=2, alpha=0.5) +
  geom_point(pch=19, size=0.7) +
 # theme_bw(base_size=10) +
  scale_x_log10(name = "H/L ratio", limits = c(10^-2, 10^2), expand=c(0,0), 
                labels=scales::comma_format(digits=1, drop0trailing=TRUE)) + 
  scale_y_log10() + 
  scale_color_manual(values=c("grey50", "darkblue"), guide=FALSE) +
  scale_alpha_manual(values=c(0.2,1), guide=FALSE) +
  theme(aspect.ratio=1,
        panel.spacing=grid::unit(1, "lines"),
        axis.text.x = element_text(angle=45, hjust = 1))
```

```
## Warning: Transformation introduced infinite values in continuous y-axis
```

```
## Warning: Removed 1 rows containing missing values (geom_point).
```

We can now see how highlighting the hyphae or yeast hits on all plots looks like. The following plot shows the on all pannels those genes that are significant in both samples in blue and those that are significant only in yeast in red:

```
ggplot(results) +
  aes(H.L.ratio, Intensity, color=sig.yeast.IP=="+", alpha=sig.yeast.IP=="+") +
  facet_grid(fraction ~ condition) +
  geom_vline(xintercept = 1, lty=2, alpha=0.5) +
  geom_point(pch=19, size=0.7) +
  geom_point(data=function(x) subset(x, sig.yeast.IP=="+" & sig.hyphae.IP==""), color="orange", size=0.5, alpha=1) +
 # theme_bw(base_size=10) +
  scale_x_log10(name = "H/L ratio", limits = c(10^-2, 10^2), expand=c(0,0), 
                labels=scales::comma_format(digits=1, drop0trailing=TRUE)) + 
  scale_y_log10() + 
  scale_color_manual(values=c("grey50", "darkblue"), guide=FALSE) +
  scale_alpha_manual(values=c(0.2,1), guide=FALSE) +
  theme(aspect.ratio=1,
        panel.spacing=grid::unit(1, "lines"),
        axis.text.x = element_text(angle=45, hjust = 1))
```

```
## Warning: Transformation introduced infinite values in continuous y-axis
```

```
## Warning: Removed 1 rows containing missing values (geom_point).
```

The next plot shows the same but in red are those genes only signicant only in hyphae…

```
ggplot(results) +
  aes(H.L.ratio, Intensity, color=sig.hyphae.IP=="+", alpha=sig.hyphae.IP=="+") +
  facet_grid(fraction ~ condition) +
  geom_vline(xintercept = 1, lty=2, alpha=0.5) +
  geom_point(pch=19, size=0.7) +
  geom_point(data=function(x) subset(x, sig.yeast.IP=="" & sig.hyphae.IP=="+"), 
             color="red", size=0.6, pch=19) +
  theme_bw(base_size=10) +
  scale_x_log10(name = "H/L ratio", limits = c(10^-2, 10^2), expand=c(0,0), 
                labels=scales::comma_format(digits=1, drop0trailing=TRUE)) + 
  scale_y_log10() + 
  scale_color_manual(values=c("grey50", "darkblue"), guide=FALSE) +
  scale_alpha_manual(values=c(0.2,1), guide=FALSE) +
  theme(aspect.ratio=1,
        panel.spacing=grid::unit(1, "lines"),
        axis.text.x = element_text(angle=45, hjust = 1))
```

```
## Warning: Transformation introduced infinite values in continuous y-axis
```

```
## Warning: Removed 1 rows containing missing values (geom_point).
```

Lets have a look where the significant lysate genes fall:

```
results %>%
  mutate(filtered=(condition=="yeast" & sig.yeast.lysate=="+") | (condition=="hyphae" & sig.hyphae.lysate=="+")) %>%
  ggplot() +
  aes(H.L.ratio, Intensity, color=filtered, alpha=filtered) +
  facet_grid(fraction ~ condition) +
  geom_vline(xintercept = 1, lty=2, alpha=0.5) +
  geom_point(pch=19, size=0.7) +
  theme_bw(base_size=10) +
  scale_x_log10(name = "H/L ratio", limits = c(10^-2, 10^2), expand=c(0,0), 
                labels=scales::comma_format(digits=1, drop0trailing=TRUE)) + 
  scale_y_log10() + 
  scale_color_manual(values=c("grey50", "darkblue"), guide=FALSE) +
  scale_alpha_manual(values=c(0.2,1), guide=FALSE) +
  theme(aspect.ratio=1,
        panel.spacing=grid::unit(1, "lines"),
        axis.text.x = element_text(angle=45, hjust = 1))
```

```
## Warning: Transformation introduced infinite values in continuous y-axis
```

```
## Warning: Removed 1 rows containing missing values (geom_point).
```

We can also mark those that are filtered due to the lysate and significant by the IP in red)

```
results %>%
  subset(fraction=="IP") %>%
  mutate(filtered=(condition=="yeast" & sig.yeast.lysate=="+") | (condition=="hyphae" & sig.hyphae.lysate=="+")) %>%
  ggplot() +
  aes(H.L.ratio, Intensity, color=filtered, alpha=filtered) +
  facet_grid(fraction ~ condition) +
  geom_vline(xintercept = 1, lty=2, alpha=0.5) +
  geom_point(pch=19, size=0.7) +
  geom_point(data=function(x) subset(x, filtered & significant=="+"), color="red", size=0.5) +
  theme_bw(base_size=10) +
  scale_x_log10(name = "H/L ratio", limits = c(10^-2, 10^2), expand=c(0,0), 
                labels=scales::comma_format(digits=1, drop0trailing=TRUE)) + 
  scale_y_log10() + 
  scale_color_manual(values=c("grey50", "darkblue"), guide=FALSE) +
  scale_alpha_manual(values=c(0.2,1), guide=FALSE) +
  theme(aspect.ratio=1,
        panel.spacing=grid::unit(1, "lines"),
        axis.text.x = element_text(angle=45, hjust = 1))
```

```
## Warning: Removed 1 rows containing missing values (geom_point).
```

We can also look at the plots after removing the genes significant in the lysate. Yeast significant genes first:

```
library(Hmisc)
```

```
## Loading required package: lattice
```

```
## Loading required package: survival
```

```
## Loading required package: Formula
```

```
## 
## Attaching package: 'Hmisc'
```

```
## The following objects are masked from 'package:dplyr':
## 
##     combine, src, summarize
```

```
## The following objects are masked from 'package:base':
## 
##     format.pval, round.POSIXt, trunc.POSIXt, units
```

```
fancy_scientific <- function(l) {
     # turn in to character string in scientific notation
     l <- format(l, scientific = TRUE)
     # quote the part before the exponent to keep all the digits
     l <- gsub("^(.*)e", "'\\1'e", l)
     # turn the 'e+' into plotmath format
     l <- gsub("e", "%*%10^", l)
     # return this as an expression
     parse(text=l)
}
results %>%
  subset((condition=="yeast" & !sig.yeast.lysate=="+") | (condition=="hyphae" & !sig.hyphae.lysate=="+")) %>%
ggplot() +
  aes(H.L.ratio, Intensity, color=sig.yeast.IP=="+", alpha=sig.yeast.IP=="+") +
  facet_grid(fraction ~ condition, labeller=labeller(condition=capitalize, fraction=capitalize)) +
  geom_vline(xintercept = 1, lty=2, alpha=0.5) +
  geom_point(pch=19, size=0.7) +
  geom_point(data=function(x) subset(x, sig.yeast.IP=="+" & sig.hyphae.IP!="+"), color="yellow2", size=0.6, pch=19) +
    geom_point(data=function(x) subset(x, sig.yeast.IP=="+" & sig.hyphae.IP=="+"), color="darkblue", size=0.7, pch=19) +
  theme_grey(base_size=8.5) +
  scale_x_log10(name = "Heavy:Light ratio", limits = c(10^-2, 10^2), expand=c(0,0), 
                labels=scales::comma_format(digits=1, drop0trailing=TRUE)) + 
  scale_y_log10(labels=fancy_scientific) + 
  scale_color_manual(values=c("grey50", "darkblue"), guide=FALSE) +
  scale_alpha_manual(values=c(0.2,1), guide=FALSE) +
  theme(aspect.ratio=1,
        panel.spacing=grid::unit(1, "lines"),
        axis.text = element_text(size=8.5),
        axis.title = element_text(size=8.5),
        strip.text = element_text(size=8.5),
        axis.text.x = element_text(angle=45, hjust = 1)) -> g
ggsave("figs/fig2B.png", g, width=4, height=4, dpi=300)
```

```
## Warning: Transformation introduced infinite values in continuous y-axis
```

```
## Warning: Removed 1 rows containing missing values (geom_point).
```

```
print(g)
```

```
## Warning: Transformation introduced infinite values in continuous y-axis

## Warning: Removed 1 rows containing missing values (geom_point).
```

```
results %>%
  subset((condition=="yeast" & !sig.yeast.lysate=="+") | (condition=="hyphae" & !sig.hyphae.lysate=="+")) %>% group_by(sig.yeast.IP !="+", sig.hyphae.IP=="+", condition, fraction) %>% summarise(count=n())
```

Now highlighting the genes significant in hyphae:

```
results %>%
  subset((condition=="yeast" & !sig.yeast.lysate=="+") | (condition=="hyphae" & !sig.hyphae.lysate=="+")) %>%
  ggplot() +
  aes(H.L.ratio, Intensity, color=sig.hyphae.IP=="+", alpha=sig.hyphae.IP=="+") +
  facet_grid(fraction ~ condition) +
  geom_vline(xintercept = 1, lty=2, alpha=0.5) +
  geom_point(pch=19, size=0.7) +
  geom_point(data=function(x) subset(x, sig.yeast.IP=="" & sig.hyphae.IP=="+"), color="red", size=0.6, pch=19) +
  theme_bw(base_size=10) +
  scale_x_log10(name = "H/L ratio", limits = c(10^-2, 10^2), expand=c(0,0), 
                labels=scales::comma_format(digits=1, drop0trailing=TRUE)) + 
  scale_y_log10() + 
  scale_color_manual(values=c("grey50", "darkblue"), guide=FALSE) +
  scale_alpha_manual(values=c(0.2,1), guide=FALSE) +
  theme(aspect.ratio=1,
        panel.spacing=grid::unit(1, "lines"),
        axis.text.x = element_text(angle=45, hjust = 1))
```

```
## Warning: Transformation introduced infinite values in continuous y-axis
```

```
## Warning: Removed 1 rows containing missing values (geom_point).
```

So the conclusions of this exploritory data analysis (EDA) is that the yeast only and hyphae only genes are genuinely seperate. If you look that the plot that shows the yeast only genes on the hyphae IP data, you’ll see that they look more or less evenly distributed, and do not look like they are showing a paritcular enrichment in the hyphae lysate either. This will be followed in due course by a statistical analysis of these things.

## Non-detected genes

How many genes are detected in each sample

```
results %>% 
  group_by(condition, fraction) %>%
  summarise(n=n_distinct(Protein.IDs))
```

So similar numbers are detected in each experiment expect for the hyphae IP where slightly fewer are detect. How many of the yeast only genes simply arn’t detected in the hyphae IP? There are, however, 300 genes dectected in the yeast IP that aren’t in the yeast lysate. Must be careful of these genes.

The following table shows what the status of the genes that were significant in the yeast sample and not filtered out:

```
per_gene_significance %>%
  filter(sig.yeast.IP == "+",  !(!is.na(sig.yeast.lysate) & sig.yeast.lysate == "+")) %>%
  with(table(IP=sig.hyphae.IP, Lysate=sig.hyphae.lysate, useNA="ifany"))
```

```
##              Lysate
## IP                + Not Present
##               21  3          14
##   +            0  5          33
##   Not Present  6  3          32
```

The above shows that the majority of the yeast hits were simply not measured in the hyphae lysate. In the few cases where they are measured in the hyphae lysate and are significant in the hyphae IP they are also significant in the hyphae lysate. **There are no genes that are signigicant in yeast that are also significant hyphae IP, but insignificant in hyphae lysate (rather than just not present in the lysate).** This makes me wonder about the whether the yeast hits are not being filtered betcause they are not signigicant in the yeast lysate, or because they are not measured in the yeast lysate.

```
per_gene_significance %>%
  filter(sig.yeast.IP == "+") %>%
  with(table(sig.yeast.lysate, useNA="ifany"))
```

```
## sig.yeast.lysate
##                       + Not Present 
##          39          16          78
```

This suggests that most of the yeast hit genes are not classed as significant in the yeast lysate, not because they are close to zero ratio in the lysate, but because they are not detected.

Looks like we could really do with a 4 way venn diagram here of the detected proteins.

```
library(VennDiagram)
```

```
## Loading required package: grid
```

```
## Loading required package: futile.logger
```

```
library(grid)
venn <- venn.diagram(list("yeast IP"=yeast_IP$Protein.IDs,
                          "yeast lysate"=yeast_lysate$Protein.IDs,
                          "hyphal IP"=hyphae_IP$Protein.IDs,
                          "hypha lysate"=hyphae_lysate$Protein.IDs),
                     filename=NULL
                     )
grid.newpage()
grid.draw(venn)
```

What happens if we restrict this to only look at the those proteins significant in the yeast IP

```
yeast_sig <- subset(yeast_IP, B.significant.FDR.0.05 == "+")$Protein.IDs
venn <- venn.diagram(list("yeast IP"=intersect(yeast_sig,yeast_IP$Protein.IDs),
                          "yeast lysate"=intersect(yeast_sig,yeast_lysate$Protein.IDs),
                          "hyphal IP"=intersect(yeast_sig,hyphae_IP$Protein.IDs),
                          "hypha lysate"=intersect(yeast_sig,hyphae_lysate$Protein.IDs)),
                     filename=NULL
                     )
grid.newpage()
grid.draw(venn)
```

So the largest group of proteins are those that are dectected in both the IPs, but neither of the lysates. The present in all samples catgory is only just larger than the present only in yeast IP category. This makes sense I guess because IPing enriches for proteins, and without the IP they might be at too low a level to caputre. I guess what this means is that we can’t trust the lysates to definately tell us if a protein is an isotope related contaminant or not, and we should expect to find the proteins we are interested in only being present in the IP samples.

This has been a rather lengthy diversion, but I guess we now know that it is unlikely that confident, yeast only genes have been filtered out of the hyphae results because of the H/L ratios in the lysate. In fact, this category is a total of 5 genes.

So in a total of 5 genes that are signifacnt in both IPs and only the hyphal lysate, 2 are ERG genes. Note that all 5 of these genes are present in the yeast lysate as well, so it will be possible to ask if they have a higher than average H/L ratio.

## Statistical analysis

In order to look at the statistical properties of the sets of proteins, we will first define a metric. We want something that accounts for both intensity and H/L ratio. B significance does this quite well. Particularly if we take the -log10 of the B value.

The first investigation we will make is whether genes that are only significant in yeast (and not in yeast lysate) are more significant than average in hyphae.

```
library(limma)
yeast_genes = subset(per_gene_significance, sig.yeast.IP=="+" & !sig.yeast.lysate=="+" &
                       sig.hyphae.IP=="" & !sig.hyphae.lysate=="+")$Protein.IDs
results%>%
  filter(condition=="hyphae", fraction=="IP", sig.hyphae.IP=="", !sig.hyphae.lysate=="+")%>%
  with(
    barcodeplot(-log10(B.value),
                Protein.IDs %in% yeast_genes)
  )
```

```
results %>%
  filter(condition=="hyphae", fraction=="IP", sig.hyphae.IP=="", !sig.hyphae.lysate=="+") %>%
  with(
    geneSetTest(Protein.IDs %in% yeast_genes,
                -log10(B.value),
                ranks.only = TRUE))
```

```
## [1] 0.9339006
```

In the plot above, the bottom pannel shows all the proteins ranked from the least significantly enriched in the heavy fraction to the most enriched in the heavy fraction. The black lines represent the genes of interest, in this case the 35 genes that are high confidence yeast hits, but not high confidence hyphae hits, but are not filtered out because of the hyphae lysate. The line above is an average density of the black lines in that part of the ranking. As we can see, there is an enrichment of these genes at very poor signicance, but not enrichment at higher significance levels. This shows that not only are these genes not “just sub threshold”, they actaully tend to have a worst significance than the average gene.

What do they look like in the yeast lysate? The ones that are present enriched? We have to be a bit more careful here because the B value above was one sided - i.e. it was connected to whether the proteins were enirched in the heavy lysate. When we start comparing the non-IPed lysates, the Bvalues are now two-sided: i.e. enriched in *either* the heavy or light lysates. To correct for that we will multiple the -log10 B value by -1 if the H/L ratio is less than one.

```
results %>%
  mutate(metric = -log10(B.value) * if_else(H.L.ratio < 1, -1, 1)) -> results

results%>%
  filter(condition=="yeast", fraction=="lysate", !sig.yeast.lysate=="+")%>%
  with(
    barcodeplot(metric,
                Protein.IDs %in% yeast_genes)
  )
```

```
results %>%
  filter(condition=="yeast", fraction=="lysate", !sig.yeast.lysate=="+") %>%
  with(
    geneSetTest(Protein.IDs %in% yeast_genes,
                metric,
                ranks.only = TRUE))
```

```
## [1] 0.07460425
```

Interestingly here it looks like there is a slight enrichment in the light lysate for these yeast only proteins, although it is not significant.

Those yeast only hits that were filtered out of the hyphal hits, what are they doing in the yeast lyaste?

```
hfyo <- subset(per_gene_significance,
   sig.yeast.IP=="+" & sig.hyphae.IP=="+" & sig.hyphae.lysate=="+")$Protein.IDs

results%>%
  filter(condition=="yeast", fraction=="lysate", !sig.yeast.lysate=="+")%>%
  with(
    barcodeplot(metric,
                Protein.IDs %in% hfyo)
  )
```

```
results %>%
  filter(condition=="yeast", fraction=="lysate", !sig.yeast.lysate=="+") %>%
  with(
    geneSetTest(Protein.IDs %in% hfyo,
                metric,
                ranks.only = TRUE))
```

```
## [1] 0.6232061
```

So the answer is no. They are not particularly significant in the yeast lysate.

## Specific analysis of the ergostral genes

First load the ERG annotations…

```
library(tidyr)
erg_genes <- read.delim("erg_genes.tsv", header=FALSE)
names(erg_genes) <- c("ID", "Name", "aliases")
erg_genes %>% separate_rows(aliases, sep="\\|") -> erg_genes
```

Lets look at each of the sets in turn…

First yeast IP

```
results%>%
  filter(condition=="yeast", fraction=="IP")%>%
  with(
    barcodeplot(-log10(B.value),
                Protein.IDs %in% erg_genes$aliases,
                xlab=expression(paste(-log[10], "(B Significance) in yeast IP")))
  )
```

```
results %>%
  filter(condition=="yeast", fraction=="IP") %>%
  with(
    geneSetTest(Protein.IDs %in% erg_genes$aliases,
                -log10(B.value),
                ranks.only = TRUE))
```

```
## [1] 0.0007255415
```

Really strong enrichment. What about the yeast lysate…

```
results%>%
  filter(condition=="yeast", fraction=="lysate")%>%
  with(
    barcodeplot(-log10(B.value),
                Protein.IDs %in% erg_genes$aliases,
                xlab=expression(paste(-log[10], "(B Significance) in yeast lysate")))
  )
```

```
results %>%
  filter(condition=="yeast", fraction=="lysate") %>%
  with(
    geneSetTest(Protein.IDs %in% erg_genes$aliases,
                -log10(B.value),
                ranks.only = TRUE))
```

```
## [1] 0.3008014
```

No enirchment there. Okay, now the hyphae…

```
results%>%
  filter(condition=="hyphae", fraction=="IP")%>%
  with(
    barcodeplot(-log10(B.value),
                Protein.IDs %in% erg_genes$aliases,
                xlab=expression(paste(-log[10], "(B Significance) in hyphal IP")))
  )
```

```
results %>%
  filter(condition=="hyphae", fraction=="IP") %>%
  with(
    geneSetTest(Protein.IDs %in% erg_genes$aliases,
                -log10(B.value),
                ranks.only = TRUE))
```

```
## [1] 0.001350851
```

Again, pretty strong enrichment. How about the hyphal lysate…

```
results%>%
  filter(condition=="hyphae", fraction=="lysate")%>%
  with(
    barcodeplot(-log10(B.value),
                Protein.IDs %in% erg_genes$aliases,
                xlab=expression(paste(-log[10], "(B Significance) in hyphal lysate")))
  )
```

```
results %>%
  filter(condition=="hyphae", fraction=="lysate") %>%
  with(
    geneSetTest(Protein.IDs %in% erg_genes$aliases,
                -log10(B.value),
                ranks.only = TRUE))
```

```
## [1] 3.894227e-07
```

This is a really strong enrichment in the hyphal lysate. If there is such a strong enrichment in the hyphal lysate, why is it not present at all in the yeast lysate, that seems weird. How many of these genes are present in each of the expriments…

```
results %>%
  filter(Protein.IDs %in% erg_genes$aliases) %>%
  group_by(condition, fraction) %>%
  summarise(n = n_distinct(Protein.IDs))
```

So 24 of the 28 erg proteins are present in the yeast lysate, so that can’t be the explaination. What happens to these erg genes in yeast cf hyphae? Are they more highly expressed in one form cf the other? Its really weird.

Lets plot them out…

```
library(ggplot2)
ggplot(results) +
  aes(H.L.ratio, Intensity) +
  facet_grid(fraction ~ condition) +
  geom_vline(xintercept = 1, lty=2, alpha=0.5) +
  geom_point(pch=19, size=0.7, color="grey50", alpha=0.2) +
  geom_point(data=function(x) subset(x, Protein.IDs %in% erg_genes$aliases), 
             color="darkblue", alpha=1, size=0.8, pch=19) +
  theme_bw(base_size=10) +
  scale_x_log10(name = "H/L ratio", limits = c(10^-2, 10^2), expand=c(0,0), 
                labels=scales::comma_format(digits=1, drop0trailing=TRUE)) + 
  scale_y_log10() + 
  theme(aspect.ratio=1,
        panel.spacing=grid::unit(1, "lines"),
        axis.text.x = element_text(angle=45, hjust = 1))
```

```
## Warning: Transformation introduced infinite values in continuous y-axis
```

```
## Warning: Removed 1 rows containing missing values (geom_point).
```
