# Supplementary material for: Proteins that physically interact with the phosphatase Cdc14 in Candida albicans have diverse roles in the cell cycle
Source: Sci Rep. 2019 Apr 18;9:6258. doi: 10.1038/s41598-019-42530-1 (PMC6472416; doi:10.1038/s41598-019-42530-1)
Supplement: Supplementary file 6 — Proteins that physically interact with the phosphatase Cdc14 in Candida albicans have diverse roles in the cell cycle [file 41598_2019_42530_MOESM6_ESM.pdf]

Supplementary Information

Proteins that physically interact with the phosphatase Cdc14 in *Candida albicans* have diverse roles in the cell cycle

Iliyana N. Kaneva[1,2], Ian M. Sudbery [2], Mark J. Dickman*[1] Peter E. Sudbery*[2]

[1]ChELSI Institute, Department of Chemical and Biological Engineering, University of Sheffield, UK

[2] Department of Molecular Biology and Biotechnology, University of Sheffield, S10 2TN, UK

*Corresponding authors:

Contact information:

Iliyana Kaneva: iliyana.kaneva@googlemail.com

Ian Sudbery: I.Sudbery@shef.ac.uk

Mark Dickman: M.Dickman@shef.ac.uk Tel +44 114 2227541

Peter Sudbery: P.Sudbery@Shef.ac.uk Tel +44 114 2337444

Supplementary Figure S1 Full length autoradiograms of the cropped panels shown in figure 1.

a: Samples were withdrawn at the indicated time in minutes after re-inoculation of a stationary phase culture into yeast or hyphal growth conditions. Lysates were fractionated by SDS gel electrophoresis and transferred to membrane by electroblotting. NEB Biolabs Prestained broad range protein markers were used for molecular weight markers which were also transferred and were visible on the membrane after blotting. The membrane was cut horizontally at the 46 kDa marker. The upper portion was probed with the α-Myc monoclonal antibody and the lower portion with the α-PSTAIRE monoclonal antibody as a loading control. The membranes portions were then rejoined and imaged together by ECL. After the chemiluminescence had been recorded the membranes were imaged with white light to record the migration of the molecular weight markers (mw). Specificity of the monoclonal antibodies is shown by the absence of a band in the negative controls (-ve), which were derived from parental MLO4 yeast cells in mid-log phase.

Figure S1b Full length autoradiograms of the cropped panels shown in figure 1b. A Lysate from C. albicans *cdc*14^{PD}-Myc cells was treated with λ phosphatase as indicated and the Western Blot probed with an αMyc antibody

F**igure S1c Full length autoradiograms of the cropped panels shown in figure 1c**. Cell lysates of

the indicated strains were immunoprecipitated with an αMyc antibody. The Western blot was first probed with αGFP. After development with ECL the membrane was stripped and probed with an αMyc antibody. The stripping was not complete and bands corresponding to the GFP signal are still evident on the αMyc Autoradiograms. Lanes corresponding to samples from the IP, lysate serving to monitor the input ,and the supernatant after the IP was pelleted are shown as indicated

Supplementary Fig. 2 *MET3-CDC14/cdc14^{PD}-MYC* cells were grown as yeast hyphae under *MET3*-repressing conditions. Yeast cells fail to separate and hyphal cells fail to form the long thin tubes characteristic of hyphal growth

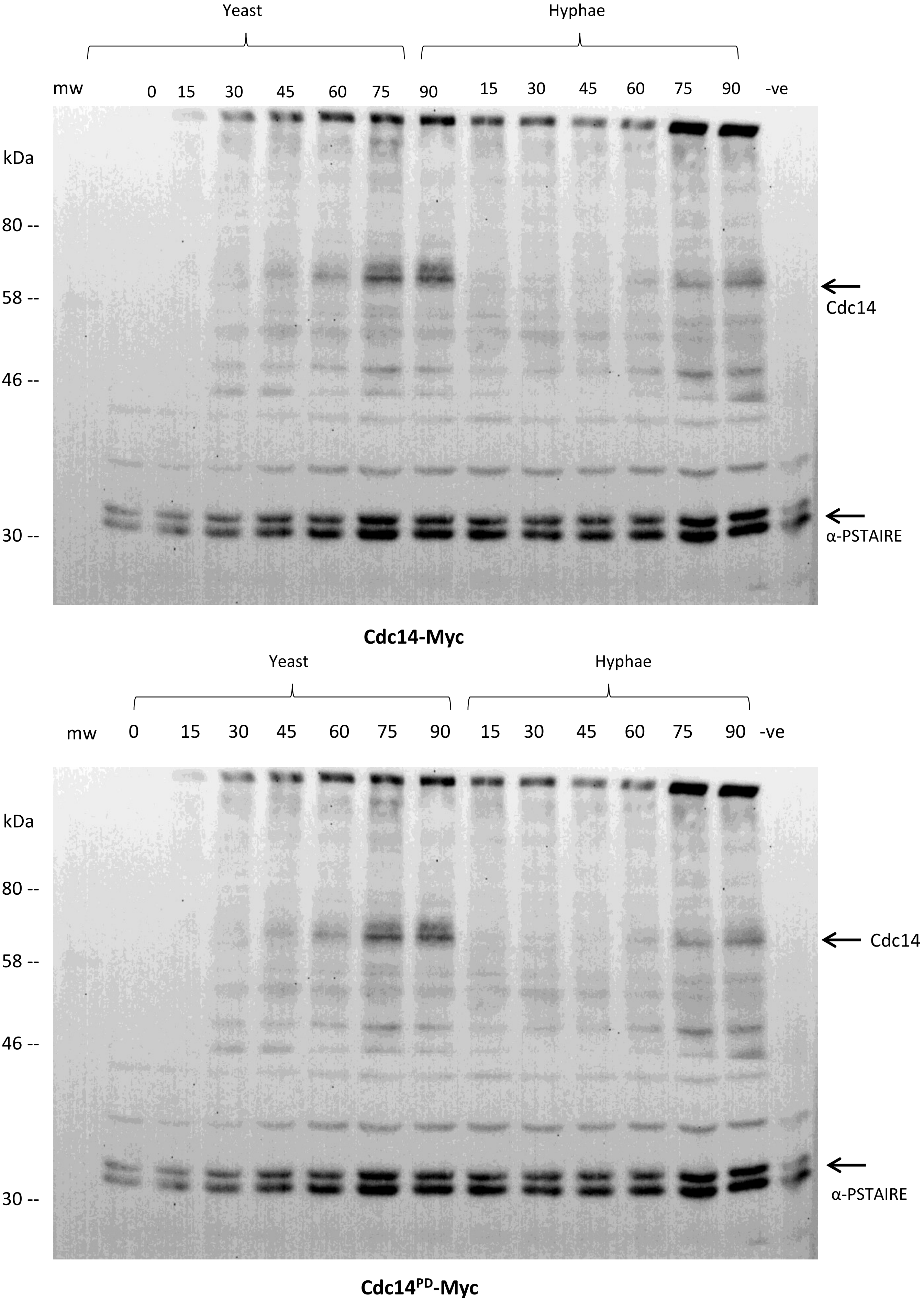

Kaneva et al Fig. S1a

Figure S1a Full length autoradiograms of the cropped panels shown in figure 1a. NEB Biolabs Prestained broad range protein markers were used for molecular weight markers which were transferred to the membrane during the Western blotting. a:The membrane was cut horizontally at the 46 kDa marker. The upper portion was probed with the α-Myc monoclonal antibody and the lower portion with the α-PSTAIRE antibody as a loading control. The membranes portions were then rejoined and imaged together by ECL. After the ECL luminescence had been recorded the membranes were imaged with white light to record the migration of the molecular weight markers (mw). Negative controls were derived from mid-log phase parental MLO4 yeast cells. Specificity of the monoclonal antibodies is shown by the absence of a band in the negative controls (-ve) which were derived from parental MLO4 yeast cells in mid-log phase.

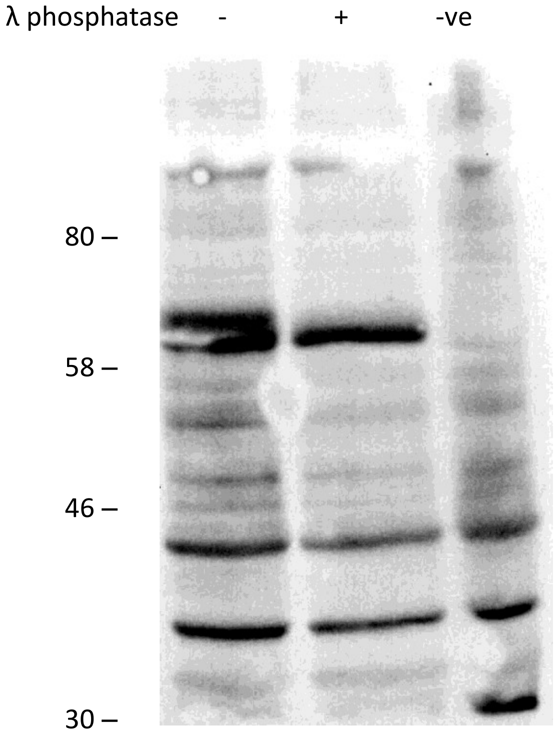

Kaneva et al Figure S1b

Figure S1b Full length autoradiograms of the cropped panels shown in figure 1b
A Lysate from *C. albicans* Cdc14^{PD}-Myc cells was treated with λ phosphatase and the Western blot probed with an αMyc antibody

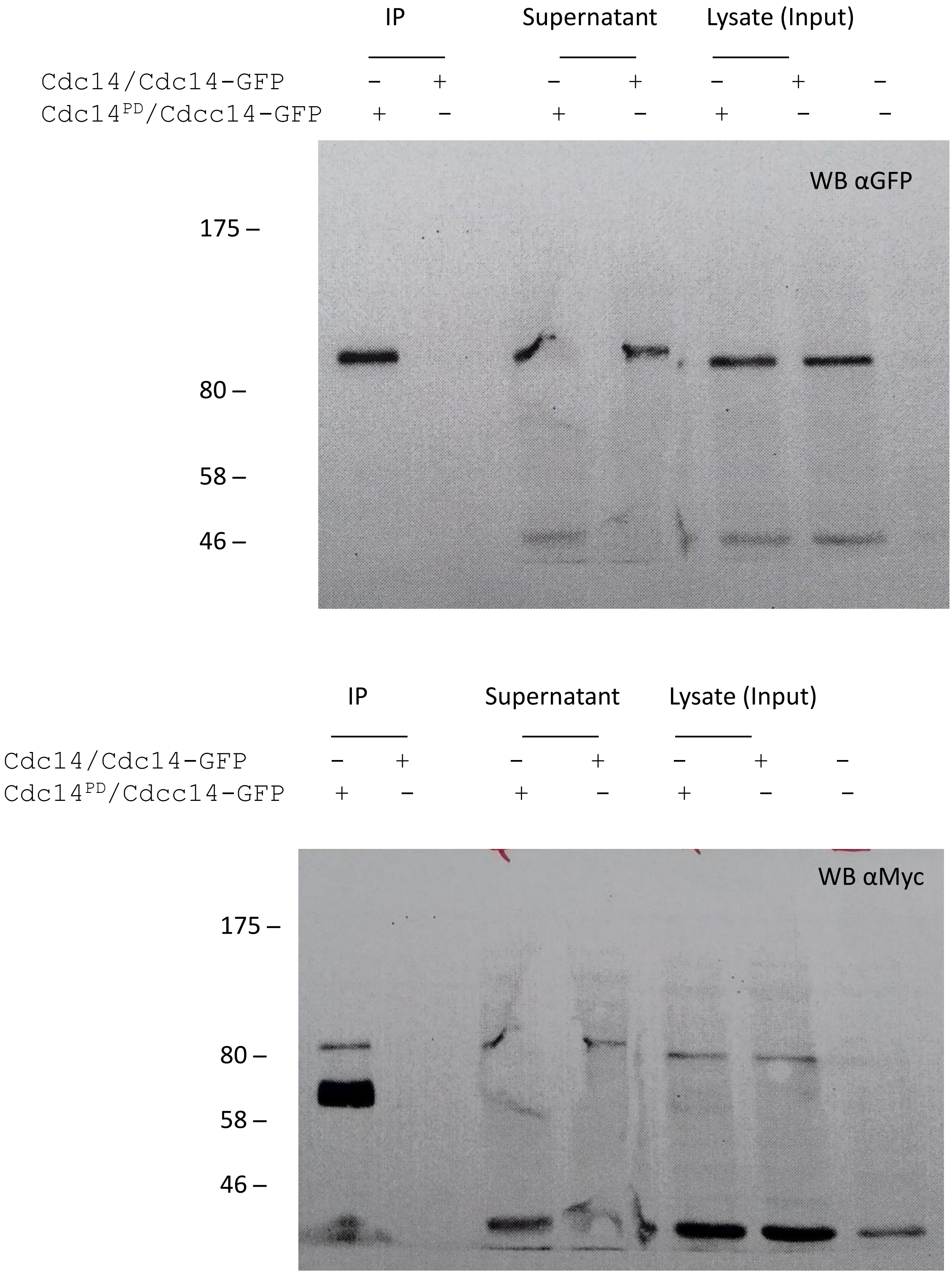

Figure S1b Full length autoradiograms of the cropped panels shown in figure 1c. Cell lysates of the indicated strains were immunoprecipitated with an αMyc antibody. The Western blot was first probed with αGFP. After development with ECL the membrane was stripped and probed with an αMyc antibody. The stripping was not complete and bands corresponding to the GFP signal are still evident on the αMyc Autoradiograms. Lanes corresponding to samples from the IP, lysate serving to monitor the input ,and the supernatant after the IP was pelleted are shown as indicated

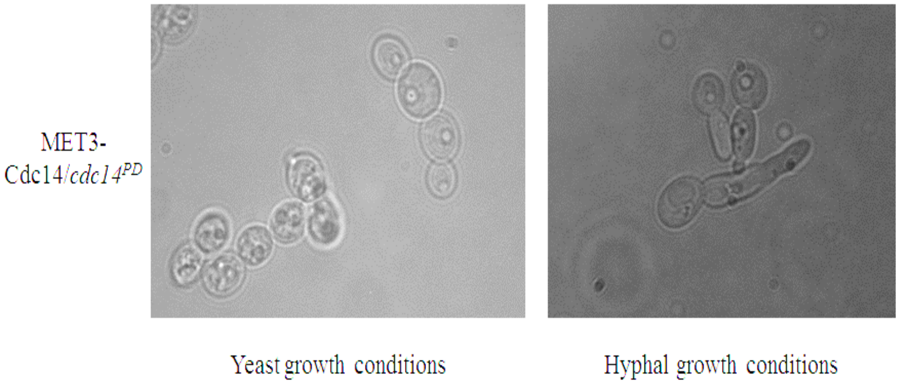

Supplementary Fig. S2 *MET3-CDC14/cdc14^{PD}-MYC* cells were grown as yeast hyphae under *MET3*-repressing conditions. Yeast cells fail to separate and hyphal cells fail to form the long thin tubes characteristic of hyphal growth

Supplementary Table S1 126 Cdc14-interacting proteins

Common Gene name	Protein_IDs	Yeast hit	Hyphae hit	Description
	orf19.2381	+	+	Protein of unknown function; possibly an essential gene, disruptants not obtained by UAU1 method; no domains
	orf19.2826	+	+	No domains
	orf19.3091	+		No domains
	orf19.3296	+		No domains
	orf19.3362/orf 19.2671*	+		Has domain(s) with predicted flavin adenine dinucleotide binding, oxidoreductase activity and role in oxidation-reduction process
	orf19.4101	+		No domains
	orf19.520	+		No domains
	orf19.5246	+		No domains
	orf19.5491	+	+	ADIP domain Afadin- and alpha -actinin-Binding; This family is found in mammals where it is localized at cell-cell adherens junctions, and in Sch. pombe and other fungi where it anchors spindle-pole bodies to spindle microtubules. It is a coiled-coil structure, and in pombe, it is required for anchoring the minus end of spindle microtubules to the centrosome equivalent, the spindle-pole body
	orf19.5518	+	+	No domains
	orf19.557	+		No domains
	orf19.6030	+		No domains
	orf19.6583	+	+	No domains
	orf19.7060	+		No domains
	orf19.7406	+		No domains
ACD99	orf19.7288	+		Protein with predicted oxidoreductase and dehydrogenase domains
ACE2	orf19.6124	+	+	Transcription factor; similar to S. cerevisiae Ace2 and Swi5; regulates morphogenesis, cell separation, adherence, virulence in a mice; mutant is hyperfilamentous
ACO1	orf19.6385	+		Aconitase; induced in high iron; 2 upstream CCAAT motifs; amino acid starvation (3-AT),

				amphotericin B, phagocytosis, farnesol induced; Hap43, fluconazole-repressed; Gcn4-regulated
ANT1	orf19.6254	+		Peroxisomal adenine nucleotide transporter; role in beta-oxidation of medium-chain fatty acid and peroxisome proliferation;
ARG1	orf19.7469	+		Argininosuccinate synthase; arginine synthesis; Gcn4, Rim101 regulated
ASK1	orf19.4675	+	+	Essential subunit of the Dam1 (DASH) complex, which acts in chromosome segregation by coupling kinetochores to spindle microtubules
BAS1	orf19.3809	+		Putative Myb-like transcription factor; ortholog S. cerevisiae Bas1, a regulator of purine biosynthetic genes; mutant exhibits adenine auxotrophy and abnormal colony morphology
BIR1	orf19.643		+	Subunit of chromosomal passenger complex (CPC); CPC is comprised of Ipl1p-Sli15p-Bir1p-Nbl1p and regulates chromosome segregation; required for chromosome bi-orientation and for spindle assembly checkpoint activation upon reduced sister kinetochore tension; relative distribution to shortened microtubules increases upon DNA replication stress; sumoylated in an Mms21p-dependent manner; human survivin homolog
CBR1	orf19.1801	+		Putative cytochrome B5 reductase; plasma membrane-localized
CDC16	orf19.1792	+		Subunit of the anaphase-promoting complex/cyclosome (APC/C); which is a ubiquitin-protein ligase required for degradation of anaphase inhibitors, including mitotic cyclins, during the metaphase/anaphase transition; required for sporulation; relocalizes to the cytosol in response to hypoxia
CDC27	orf19.3231	+		Putative ubiquitin-protein ligase; periodic mRNA expression, peak at cell-cycle S/G2 phase; Subunit of the Anaphase-Promoting Complex/Cyclosome (APC/C)
CDC28	orf19.3856	+		Cyclin-dependent protein kinase; interacts with regulatory subunit Cyb1; determination of cell morphology during the cell cycle
CDC5	orf19.6010	+	+	Polo-like kinase; member of conserved Mcm1 regulon; depletion causes defects in spindle elongation and Cdc35-dependent filamentation
CDC7	orf19.3561	+	+	Catalytic subunit of Dbf4p-regulated serine/threonine protein kinase; negative regulator of hyphal development; cell-cycle regulated periodic mRNA expression
CDC9	orf19.6155	+		DNA ligase I found in nucleus and mitochondria; essential enzyme that joins Okazaki fragments during DNA replication; also acts in ribonucleotide excision repair, base excision repair, and recombination; DNA ligase I mutants trigger ubiquitination of PCNA at K107, facilitating Rad59p-mediated bypass of unligated Okazaki fragments; human homolog LIG1 can complement yeast cdc9 temperature-sensitive mutant at restrictive temperature
CDH1	orf19.2084	+	+	Protein involved in regulation of mitosis; similar to S. cerevisiae Cdh1, which is an APC/C component; transcriptionally induced by Mnl1 under weak acid stress
CHT4	orf19.1515	+	+	Chitinase; similar to S. cerevisiae sporulation-specific Cts2p
CLB2	orf19.1446	+		B-type mitotic cyclin (cyclin-dependent protein kinase regulatory subunit); essential; required for

				wild-type mitotic exit; role in cell polarization; interacts with catalytic subunit Cdk1
CPA2	orf19.3221	+		Putative arginine-specific carbamoylphosphate synthetase
CSI2	orf19.3535	+		Protein of unknown function; green fluorescent protein (GFP)- fusion protein localizes to the mother side of the bud neck and the vacuole
CSM1	orf19.7663	+		Putative component of the monopolin complex with role in rDNA silencing, homologous chromosome segregation, protein localization to nucleolar rDNA repeats
DAD2	orf19.3551	+	+	Essential subunit of the Dam1 (DASH) complex, which acts in chromosome segregation by coupling kinetochores to spindle microtubules
DAD3	orf19.3871	+		Subunit of the Dam1 (DASH) complex, which acts in chromosome segregation by coupling kinetochores to spindle microtubules
DAK2	orf19.4777	+		Putative dihydroxyacetone kinase; repressed by yeast-hypha switch
DAL81	orf19.3252	+		Zn(II)2Cys6 transcription factor; ortholog of S. cerevisiae Dal81, involved in the regulation of nitrogen-degradation genes; required for yeast cell adherence to silicone substrate
DAM1	orf19.4837	+	+	Essential subunit of the Dam1 (DASH) complex, which acts in chromosome segregation by coupling kinetochores to spindle microtubules
DBF2	orf19.1223	+		Essential serine/threonine protein kinase involved in mitotic spindle formation and cytokinesis; required for septum formation, exit from mitosis, and normal hyphal morphogenesis
DBF4	orf19.5166	+	+	
DCI1	orf19.6443	+		Protein with an enoyl-CoA hydratase related domain
DUO1	orf19.1428	+	+	Subunit of the Dam1 (DASH) complex, which acts in chromosome segregation by coupling kinetochores to spindle microtubules
EHT1	orf19.3040	+		Putative acyl-coenzymeA:ethanol O-acyltransferase; regulated by Sef1, Sfu1, and Hap43
ERG1	orf19.406	+		Squalene epoxidase, catalyzes epoxidation of squalene to 2,3(S)-oxidosqualene in the ergosterol biosynthesis pathway
ERG2	orf19.6026	+		Verified ORF; C-8 sterol isomerase; enzyme of ergosterol biosynthesis pathway; converts fecosterol to episterol.
ERG24	orf19.1598	+		C-14 sterol reductase, has a role in ergosterol biosynthesis;
ERG26	orf19.2909	+		C-3 sterol dehydrogenase, catalyzes the second of three steps required to remove two C-4 methyl groups from an intermediate in ergosterol biosynthesis
ERG27	orf19.3240	+		3-Keto sterol reductase of ergosterol biosynthesis;acts in C-4 sterol demethylation with Erg25p and Erg26p
ERG6	orf19.1631	+		Delta(24)-sterol C-methyltransferase, converts zymosterol to fecosterol in ergosterol biosynthesis
ERG7	orf19.1570	+		2,3-epoxysqualene-lanosterol cyclase (lanosterol synthase), conversion of 2,3-oxidosqualene to lanosterol in sterol biosynthesis

ESP1	orf19.3356	+		Putative caspase-like cysteine protease; mutation confers increased sensitivity to nocodazole; periodic mRNA expression, peak at cell-cycle S/G2 phase; mRNA binds She3; Separase, a caspase-like cysteine protease; promotes sister chromatid separation by mediating dissociation of the cohesin Scc1p from chromatin; inhibits protein phosphatase 2A-Cdc55p to promote mitotic exit
EXO1	orf19.926	+		Putative exodeoxyribonuclease; cell-cycle regulated periodic mRNA expression
FAA21	orf19.272	+		Predicted acyl CoA synthetase; upregulated upon phagocytosis; transcript regulated by Nrg1 and Mig1
FCY21	orf19.1357	+		High affinity, high capacity, hypoxanthine-adenine-guanine-cytosine/H+ symporter
FDH1	orf19.638	+		Formate dehydrogenase; oxidizes formate to CO2; Mig1 regulated; induced by macrophages; fluconazole-repressed; repressed by Efg1 in yeast, not hyphal conditions
FKH2	orf19.5389	+	+	Forkhead transcription factor; morphogenesis regulator; required for wild-type hyphal transcription, cell separation, and for virulence in cell culture
FMA1	orf19.6837	+		Putative oxidoreductase
FUN30	orf19.6291	+		Ortholog(s) have DNA binding, DNA-dependent ATPase activity, chromatin binding activity
GDH3	orf19.4716	+		NADP-glutamate dehydrogenase; Nrg1, Plc1 regulated
GFA1	orf19.1618	+		Glucosamine-6-phosphate synthase, homotetrameric enzyme of chitin/hexosamine biosynthesis
GIN1	orf19.658	+	+	Protein involved in regulation of DNA-damage-induced filamentous growth; putative component of DNA replication checkpoint; ortholog of S. cerevisiae Mrc1p, an S-phase checkpoint protein
GLT1	orf19.6257	+		Putative glutamate synthase; regulated by Sef1, Sfu1, and Hap43
GRE24	orf19.6868	+		Putative NADPH-dependent methylglyoxal reductase; homozygous transposon insertion causes decreased colony wrinkling under filamentous growth-inducing conditions, but does not block true hyphal formation in liquid media
HAM1	orf19.1108		+	Putative deoxyribonucleoside triphosphate pyrophosphohydrolase; caspofungin repressed; regulated by Gcn2p and Gcn4p
HAT2	orf19.7185	+		Subunit of the Hat1p-Hat2p histone acetyltransferase complex; required for high affinity binding of the complex to free histone H4, thereby enhancing Hat1p activity; similar to human RbAp46 and 48; has a role in telomeric silencing
IDP2	orf19.3733	+		Isocitrate dehydrogenase; white-opaque switch regulated; morphology-regulation by Ssn6; protein in exponential and stationary phase yeast; Hap43-repressed; Spider biofilm repressed by Bcr1, Tec1, Ndt80, Rob1, Brg1; Spider biofilm induced
IFD3	orf19.3311	+		Putative aldo/keto reductase; Mig1-regulated
IPI3	orf19.6234		+	Putative U2 snRNP component
IPL1	orf19.3474		+	Putative Aurora kinase; Hap43-induced

IQG1	orf19.6536	+		Actomyosin ring component at bud neck; cell-cycle regulated ser phosphorylation at CDK sites regulate association with Bni1/Bnr1, Iqg1 degradation, and ring disassembly; mutation causes cytokinetic defects
KRI1	orf19.1609		+	Essential nucleolar protein required for 40S ribosome biogenesis; associate with snR30; physically and functionally interacts with Krr1p
LPG20	orf19.771	+		Aldo-keto reductase family protein; similar to aryl alcohol dehydrogenases
MCR1	orf19.3507	+		NADH-cytochrome-b5 reductase; soluble in hyphae
MED1	orf19.826	+		RNA polymerase II mediator complex subunit; RNA polymerase II transcription cofactor
MLC1	orf19.2416.1	+		Microtubule-dependent localized protein; at Spitzenkorper and cytokinetic ring in hyphae; cell-cycle dependent localization to tip polarisome, bud neck in yeast and pseudohyphae
MSB1	orf19.1133		+	Putative regulator of transcription; expression in S. cerevisiae flo8 and flo11 mutants suggests a role in regulation of adhesion factors
NCB2	orf19.5825		+	Beta subunit of NC2, heterodimeric regulator of transcription; activates CDR1 transcription
NCP1	orf19.2672	+		NADPH-cytochrome P450 reductase, acts with Erg11p in sterol 14 alpha-demethylation in ergosterol biosynthesis
NDC80	orf19.2827	+	+	Component of the kinetochore-associated Ndc80 complex; conserved coiled-coil protein involved in chromosome segregation, spindle checkpoint activity, and kinetochore assembly and clustering; evolutionarily conserved; complex members include Ndc80p, Nuf2p, Scp24p, and Spc25p; modified by sumoylation
NET1	orf19.267	+	+	Core subunit of the RENT complex; involved in nucleolar silencing and telophase exit; stimulates transcription by RNA polymerase I and regulates nucleolar structure
NUF2	orf19.1941	+		Kinetochore component; amount of Nuf2p and Mtw1p protein detected at each centromere is consistent with a single kinetochore microtubule attachment site
ORC1	orf19.3000	+	+	
ORC2	orf19.5358	+	+	
ORC3	orf19.6942	+	+	
ORC4	orf19.4221	+	+	
ORC5	orf19.2369	+		
ORC6	orf19.3289	+		
OSM1	orf19.6882	+		Putative flavoprotein subunit of fumarate reductase
PSD2	orf19.3954	+		Ortholog(s) have phosphatidylserine decarboxylase activity, role in phosphatidylcholine biosynthetic process and cell division site, cytosol, endosome localization
PUS1	orf19.3477	+		Putative pseudouridine synthase; predicted role in snRNA pseudouridine synthesis, tRNA

				pseudouridine synthesis
RAD52	orf19.4208	+	+	Required for homologous DNA recombination, repair of UV- or MMS-damaged DNA, telomere length, UV-induced LOH; constitutive expression, MMS-induced; weakly complements S. cerevisiae rad52 mutant
RAD9	orf19.4275	+	+	DNA damage-dependent checkpoint protein; involved in regulation of DNA-damage-induced filamentous growth
RHR2	orf19.5437	+		Glycerol 3-phosphatase; roles in osmotic tolerance, glycerol accumulation in response to salt; Spider/flow model biofilm induced; regulated by macrophage, stress, yeast-hyphal switch, pheromone, Gcn4, Hog1, Nrg1, Tup1
RIF1	orf19.427	+	+	Ortholog(s) have telomeric DNA binding activity, role in DNA double-strand break processing, DNA replication initiation, chromatin silencing at silent mating-type cassette, telomere capping and shelterin complex localization
RNH1	orf19.5614		+	Putative ribonuclease H1
RTC4	orf19.580	+		Protein of unknown function; mutants are viable
SAE2	orf19.4988	+	+	S. cerevisiae ortholog Sae2 is an endonuclease that processes hairpin DNA structures with the MRX complex, involved in meiotic and mitotic double-strand break repair; upregulated in a cyr1 null mutant
SIN3	orf19.6011	+		Protein similar to S. cerevisiae Sin3p (transcriptional corepressor involved in histone deacetylase recruitment); has paired amphipathic helix PAH1 domain; interacts with ScOpi1p, not CaOpi1p; transposon mutation affects filamentous growth
SLD2	orf19.2389	+		Single-stranded DNA origin-binding and annealing protein; required for initiation of DNA replication; phosphorylated in S phase by cyclin-dependent kinases (Cdks), promoting origin binding, DNA replication and Dpb11p complex formation; component of the preloading complex; binds the Mcm2-7p complex to prevent inappropriate Mcm2-7p interaction with the GINS complex in G1; required for S phase checkpoint; relative distribution to the nucleus increases upon DNA replication stress
SLI15	orf19.6049	+	+	Subunit of the conserved chromosomal passenger complex (CPC); complex regulates kinetochore-microtubule attachments, activation of the spindle tension checkpoint, and mitotic spindle disassembly; other complex members are Ipl1p, Bir1p, and Nbl1p
SLK19	orf19.2684	+	+	SMC_N Superfamily domain involved in Chromosome segregation ATPase [Cell cycle control, cell division, chromosome partitioning]
SLM1	orf19.4043	+		Phosphoinositide PI4,5P(2) binding protein, forms a complex with Slm2p; acts downstream of Mss4p in a pathway regulating actin cytoskeleton organization in response to stress; TORC2 complex substrate and effector; protein abundance increases in response to DNA replication stress; SLM1 has a paralog, SLM2, that arose from the whole genome duplication

SPC19	orf19.4473	+	+	Essential subunit of the Dam1 (DASH) complex, which acts in chromosome segregation by coupling kinetochores to spindle microtubules
SPC34	orf19.3788	+	+	Subunit of the Dam1 (DASH) complex, which acts in chromosome segregation by coupling kinetochores to spindle microtubules
SPS19	orf19.3684	+		Putative oxidoreductase
SQS1	orf19.2400	+		Protein that stimulates the ATPase and helicase activities of Prp43p; acts with Prp43p to stimulate 18s rRNA maturation by Nob1p; overexpression antagonizes the suppression of splicing defects by spp382 mutants; component of pre-ribosomal particles; relocalizes from nucleus to nucleolus upon DNA replication stress
SRB9	orf19.1451	+		Subunit of the RNA polymerase II mediator complex; transposon mutation affects filamentous growth
STU1	orf19.4435	+	+	Ortholog(s) have structural constituent of cytoskeleton activity, role in microtubule nucleation and spindle pole body localization
STU2	orf19.6610	+		Microtubule-associated protein (MAP) of the XMAP215/Dis1 family; regulates microtubule dynamics during spindle orientation and metaphase chromosome alignment; interacts with spindle pole body component Spc72p
THP1	orf19.5276	+		Putative nuclear pore-associated protein; Hap43p-induced gene; induced upon low-level peroxide stress; possibly an essential gene, disruptants not obtained by UAU1 method
TNA1	orf19.2397	+		High affinity nicotinic acid plasma membrane permease; responsible for uptake of low levels of nicotinic acid; expression of the gene increases in the absence of extracellular nicotinic acid or para-aminobenzoate (PABA)
TRS33	orf19.6496	+		Putative TRAPP complex subunit; constitutive expression independent of MTL or white-opaque status
YAK1	orf19.147	+		Predicted serine-threonine protein kinase; involved in hyphal growth regulation and biofilm formation
YDJ1	orf19.6408		+	Putative DnaJ-like heat shock/chaperone
YEN1	orf19.652	+	+	Holliday junction resolvase; promotes template switching during break-induced replication (BIR), causing non-reciprocal translocations (NRTs); localization is cell-cycle dependent and regulated by Cdc28p phosphorylation; homolog of human GEN1; similar to S. cerevisiae endonuclease Rth1p
YHB1	orf19.3707	+		Nitric oxide dioxygenase; acts in nitric oxide scavenging/detoxification; role in virulence in mouse; transcript activated by NO, macrophage interaction; Hap43, hypha repressed; mRNA binds She3
YHR045W	orf19.4013	+		Putative protein of unknown function; possible role in iron metabolism and/or amino acid and carbohydrate metabolism; green fluorescent protein (GFP)-fusion protein localizes to the endoplasmic reticulum
YJL068C	orf19.6596	+		Putative esterase; possibly transcriptionally regulated by Tac1; induced by Mnl1 under weak acid

				stress; protein present in exponential and stationary growth phase yeast cultures
YNK1	orf19.4311	+		Nucleoside diphosphate kinase (NDP kinase)
YPL247C	orf19.384	+		Putative protein of unknown function; green fluorescent protein (GFP)-fusion protein localizes to the cytoplasm and nucleus; similar to the petunia WD repeat protein an11; overexpression causes a cell cycle delay or arrest
YPR127W	orf19.7306	+		Aldo-keto reductase; increased transcript associated with MDR1 overexpression, benomyl or long-term fluconazole treatment; overexpression does not affect drug or oxidative stress sensitivity; stationary phase enriched
ZDS1	orf19.3823	+		Protein with a role in regulating Swe1p-dependent polarized growth; involved in maintaining Cdc55p in the cytoplasm where it promotes mitotic entry; involved in mitotic exit through Cdc14p regulation; interacts with silencing proteins at telomeres; has a role in Bcy1p localization; implicated in mRNA nuclear export

***** Orf19.336 and orf19.2671 are 98% identical and therefore could not be distinguished from each other.

Supplementary Table S2 Oligonucleotides used

Primer	Sequence in direction 5’->3’
Ask1-XFP_F	CAAAAGATTTATCAAAAGATTTAACAGAAAATTCAACTACTGATTTAGGTCCATTTAGAGAAAGATGGAAGAAATTGACAAGAAAAggtgctggcgcaggtgcttc
Ask1-XFP_R	AGGAGGCAATTATCTAACTAGAAGGTTCTAATGTTGAAACTCTTTCTTTCTTTCTTTCTTTCTTTTTGCACCCAATTAGTGATATCtctgatatcatcgatgaattcgag
Ask1_chk_F	CAATCAACTCCAAGATCGAGTG
Ask1-HA_F	ATTTAACAGAAAATTCAACTACTGATTTAGGTCCATTTAGAGAAAGATGGAAGAAATTGACAAGAAAAggtcgacggatccccgggttatacccatacgatgttcctgac
Ask1-HA_R	AGGAGGCAATTATCTAACTAGAAGGTTCTAATGTTGAAACTCTTTCTTTCTTTCTTTCTTTCTTTTTGCACCCAATTAGTGATATCtcgatgaattcgagctcgtt
S1-Cdc14-XFP	GGATTGCTTCTGGAAACTCACAAACATCAAGAGCACACTCTGGTGGTGTGAGAAAGTTAAGTGGAAAGAAACATggtgctggcgcaggtgcttc
S2-Cdc14*-XFP	CCGACTTGGCCAAGCCTAGATCCCGACTAATAGGAATTGATTTGGATGGTATAAACGGAAACAAAAAAAAGAGCTGGTACTACtctgatatcatcgatgaattcgag
S2-Cdc14-XFP	GGATTTCGATATATTGGCTTTTGCATATGGTTCGGAAGAACAAATTGAAATTGTTGAACCAGCTTATGAAGAAGACTAATTTAGtctgatatcatcgatgaattcgag
Cdc14-TAP-MYC_F	CTCACAAACATCAAGAGCACACTCTGGTGGTGTGAGAAAGTTAAGTGGAAAGAAACATggtcgacggatccccgggttagaacagaagcttatatccgaa
Cdc14-TAP-HA-MYC_R	CGAGTGGCCTATCCAAAAGATTCAACTCAGCCTTATTCCAATAACTGGATTGAATTGAGTGAAGATAGTGATATTGCTGtcgatgaattcgagctcgtt
Cdc14-chk_F	GACATCTCCACTTGCTGATTCTTCTG
Cdc14-chk-myc_R	GGCTTTTGCATATGGTTCGGAAGAAC

Cdc14-URAF_F	GTCTTTCATTCAAAAACACGTTTGTTTCTACCATACCGTTCTAAACTACCT AATAATCACAAACACCTTTTCCGctcgaggaagttcctatactttc
Cdc14-URAF_R	CGAGTGGCCTATCCAAAAGATTCAACTCAGCCTTATTCCAATAACTGGA TTGAATTGAGTGAAGATAGTGATATTGCTGctctagaactagtggatctgaagtt
NotI-Cdc14-3'_F	GGCGGG GCGGCCGC GTCTTCTTCATAAGCTGGTTCAAC
SacI-Cdc14-3'_R	GGCGGG GAGCTC CGGAGAATACAAGTACCATTCTCAAG
Cdc14-S1-MET3_F	AAATGTATATAACGAAGATGACTATCATCAATGGTCCGGTTAGTAAAGC GAACAAGCTTTATAAAAATAGTTATGCTGAACGTACCATgaagcttcgtacg ctgcaggtc
Cdc14-S2-MET3_R	AAAGGTAGAACAATCAATTTGAAGTAGATTTTCCCAACATACTTTTAAG AAACTCTATAAGAGGCACATGAACCAGTGAACTATGcatgttttctggggagg gtatttac
MET3-chk_F	GCGCCCCTCTAAAACAATACCC
Cdc14-5'chk_R	GGTAATGCGTCTTCAACTGTG
Cdc14-TAP-MYC_F	CTCACAAACATCAAGAGCACACTCTGGTGGTGTGAGAAAGTTAAGTGG AAAGAAACATggtcgacggatccccgggttagaacagaagcttatatccgaa
Cdc14-TAP-HA-MYC_R	CGAGTGGCCTATCCAAAAGATTCAACTCAGCCTTATTCCAATAACTGGA TTGAATTGAGTGAAGATAGTGATATTGCTGtcgatgaattcgagctcgtt
Cdc14-chk_F	GACATCTCCACTTGCTGATTCTTCTG
Cdc14-chk-myc_R	GGCTTTTGCATATGGTTCGGAAGAAC
Cdc14-URAF_F	GTCTTTCATTCAAAAACACGTTTGTTTCTACCATACCGTTCTAAACTACCT AATAATCACAAACACCTTTTCCGctcgaggaagttcctatactttc
Cdc14-URAF_R	CGAGTGGCCTATCCAAAAGATTCAACTCAGCCTTATTCCAATAACTGGA TTGAATTGAGTGAAGATAGTGATATTGCTGctctagaactagtggatctgaagtt
NotI-Cdc14-3'_F	GGCGGG GCGGCCGC GTCTTCTTCATAAGCTGGTTCAAC
SacI-Cdc14-3'_R	GGCGGG GAGCTC CGGAGAATACAAGTACCATTCTCAAG

Cdc14-S1-MET3_F	AAATGTATATAACGAAGATGACTATCATCAATGGTCCGGTTAGTAAAGCGAACAAGCTTTATAAAAATAGTTATGCTGAACGTACCATgaagcttcgtacgctgcaggtc
Cdc14-S2-MET3_R	AAAGGTAGAACAATCAATTTGAAGTAGATTTTCCCAACATACTTTTAAGAAACTCTATAAGAGGCACATGAACCAGTGAACTATGcatgttttctggggagggtatttac
S2-Cdc14-XFP	GGATTTCGATATATTGGCTTTTGCATATGGTTCGGAAGAACAAATTGAAATTGTTGAACCAGCTTATGAAGAAGACTAATTTAGtctgatatcatcgatgaattcgag
BamHI linker	GAT CCC TCC CAG AAC
XhoI linker	TCG AGT TCT GGG AGG
XhoI-Cdc14_F	GGC GGG CTC GAG GGC TTT CCT TTC CTT TGC TAT G
XbaI-Cdc14-MYC_R	GGC GGG TCT AGA CTA ATT TGT GAG TTT AGT ATA CAT GC
Cdc14-seq1_R	GAGGCACATGAACCAGTGAAC
Cdc14-seq2_F	GATGGAAGAGATCTTTTTGGAATTTC
Cdc14-seq3_F	CCAGAATTGGGCTCCTCATCAAG
Cdc14-seq4_F	GGTTGTTTGATTGGAGCCCATC
Cdc14-seq5_F	GCTCACCAGCAAGGTATGACTC
Cdc14 C275S F	GCAGTACATTCTAAAGCAGGGTTAGG
Cdc14 C275S R	CCTAACCCTGCTTTAGAATGTACTGC
Cdc14C275S_chk_R	CCGGTTCTTCCTAACCCTGCTTTAG

Supplementary Dataset S1 Excel file of the Statistical analysis of the peptide ratios carried out in Perseus v.1.2.5.6. Orf annotations are as downloaded from CGD. Separate worksheets are as follows

Hits lists proteins in which the H:L ratio was significantly different from a 1:1 ratio at an FDR of 0.05 after removal of proteins that were also significantly enriched in the combined lysates from the heavy and light cultures. A "+" in the yeast and/or hyphal column indicates the IP in which the H:L ratio was significantly elevated. For each morphological state, the data are combined from two biological replicates. Orf19.336 and Orf19.2671 are 98% identical and therefore could not be distinguished from each other.

Hits also significant in lysate Proteins that were also significantly elevated H:L ratio in the combined lysate before affinity purification. These proteins were removed from the initial list of proteins that showed significantly elevated HL ratio in the combined IP. Note some proteins have been removed from hyphal list but they remained the yeast list. These are encoded by orfs 19.1631, 19.1801, 19.3040, 19.3240 and 19.6837. So these 5 proteins are present in both worksheets of removed hits and confident hits.

Initial hit list shows the hits including proteins where the H:L ratio was also elevated in the combined lysate. Highlighted cells shows where the H:L ratio was significantly elevated in both the IP and combined lysate

Complete list of proteins- yeast Lists all proteins detected in the combined yeast IPs. Significance at three different FDR values are shown for each protein

Complete list of proteins- hyphae Lists all proteins detected in the combined hyphal IPs. Significance at three different FDR values are shown for each protein.

Supplementary Dataset S2 Proteins enriched in the yeast IP that were not detected in the lysate

Dataset S3 GO analysis of proteins showing significant heavy peptide enrichment in the IP. Excel file of *C. albicans* GO Term finder Process analysis using the GO Term Finder tool on the CGD website (http://www.candidagenome.org/). The query set was the list of 126 proteins in S1 Table. The default background gene set was used.

Supplementary Dataset S4 Canonical Cdc14 dephosphorylation motifs in Cdc14-interacting proteins

Dataset S5 Datasets, notebooks of statistical procedures followed and computer code used for further statistical analysis of the following

- Further verification of yeast-specific hits that are significantly enriched in the IP
- Enrichment of genes annotated as Ergosterol synthesis
- Cdc14 target sites
- Concordance with other surveys of Cdc14 interactors
- Motif enrichment in the list of 126 hit
